# High-fat diet induces C-reactive protein secretion, promoting lung adenocarcinoma via immune microenvironment modulation

**DOI:** 10.1242/dmm.050360

**Published:** 2023-11-09

**Authors:** Wei-Lun Hsu, Yun-Ting Hsieh, Wei-Ming Chen, Min-Hui Chien, Wei-Jia Luo, Jung-Hsuan Chang, Kevin Devlin, Kang-Yi Su

**Affiliations:** ^1^ Department of Clinical Laboratory Sciences and Medical Biotechnology, College of Medicine, National Taiwan University, Taipei 10055, Taiwan; ^2^ Genome and Systems Biology Degree Program, National Taiwan University and Academia Sinica, Taipei 10617, Taiwan; ^3^ Department of Laboratory Medicine, National Taiwan University Hospital, Taipei 10055, Taiwan

**Keywords:** Lung cancer, Mutant EGFR transgenic mice, High-fat diet, Tumor microenvironment, CRP

## Abstract

To understand the effects of a high-fat diet (HFD) on lung cancer progression and biomarkers, we here used an inducible mutant epidermal growth factor receptor (EGFR)-driven lung cancer transgenic mouse model fed a regular diet (RD) or HFD. The HFD lung cancer (LC-HFD) group exhibited significant tumor formation and deterioration, such as higher EGFR activity and proliferation marker expression, compared with the RD lung cancer (LC-RD) group. Transcriptomic analysis of the lung tissues revealed that the significantly changed genes in the LC-HFD group were highly enriched in immune-related signaling pathways, suggesting that an HFD alters the immune microenvironment to promote tumor growth. Cytokine and adipokine arrays combined with a comprehensive analysis using meta-database software indicated upregulation of C-reactive protein (CRP) in the LC-HFD group, which presented with increased lung cancer proliferation and metastasis; this was confirmed experimentally. Our results imply that an HFD can turn the tumor growth environment into an immune-related pro-tumorigenic microenvironment and demonstrate that CRP has a role in promoting lung cancer development in this microenvironment.

## Teaser

**Summary:** High-fat diet can elevate C-reactive protein to promote immune microenvironment modulation and tumor progression in mice with EGFR-driven lung cancer.

## INTRODUCTION

The increase in the overweight and obese population is a critical public health concern. According to the World Health Organization (WHO), ∼1 billion people were obese in 2022, including 650 million adults, 340 million adolescents and 39 million children (WHO, 2022). Obesity, as a consequence of imbalance between energy intake and energy expenditure, is caused by high-calorie uptake combined with lack of exercise. Comorbidities associated with the progression of obesity include hyperlipidemia, hypertension, cardiovascular disease, nonalcoholic fatty liver disease, chronic kidney disease, type II diabetes and cancers (GBD 2015 Obesity Collaborators et al., 2017; Lauby-Secretan et al., 2016; Lin and Li, 2021). Obesity is highly correlated with the progression and deteriorative clinical outcomes of breast, colon, liver, kidney, prostate and lung cancers (Chow et al., 2000; Hsing et al., 2007; Majed et al., 2008; Yang et al., 2017). Although the detrimental effects of obesity and overweight on health are well known from observational or epidemiological studies (Lin and Li, 2021), how a high-fat diet (HFD) is connected to cancer progression remains unclear.

Lung cancer is among the most prevalent cancer types worldwide and is a leading cause of cancer-related death in the United States, accounting for ∼350 fatalities daily (Ferlay et al., 2015; Siegel et al., 2022). Cigarette smoking is the primary etiology of lung cancer, but other risks, such as dietary factors, may also promote lung cancer over a long period (Sridhar, 1999; Alberg et al., 2013; Mithoowani and Febbraro, 2022; Herbst et al., 2018). The role of HFD in lung cancer progression is controversial. Some studies concluded that a high body mass index (BMI) was associated with reduced lung cancer risk (Mavridis and Michaelidou, 2019; Bhaskaran et al., 2014). However, according to other studies, the increase in the obese population was associated with increased lung cancer incidence rates and worse survival rates (He et al., 2017; Yu et al., 2018; Barbi et al., 2021). In animal experiments, HFD played a role in triggering pro-tumorigenic pathway activation and increased cancer metastasis through multistep processes, such as migration, invasion and angiogenesis (Scholar et al., 1989; Yan and DeMars, 2010; Shi et al., 2021; Kimura and Sumiyoshi, 2007). HFD-induced lipid droplets and adipocyte development have also been reported to support lung cancer cell growth, survival and migration (Lung et al., 2022; Li et al., 2018). Understanding the effects of HFD on lung cancer and its underlying signatures is thus crucial for improving clinical cancer management and monitoring.

Recent studies have suggested that an HFD promotes cancer progression by altering the microenvironment for cancer development. Nadella et al. (2018) reported that dietary fat stimulated cancer growth and fibrosis in the microenvironment through cholecystokinin receptors, while Tang et al. (2023) found that an HFD in early life altered Wnt signaling pathways in the mammary microenvironment, which further adjusted the tumor environment into an inflammatory state, contributing to breast cancer tumorigenesis. Furthermore, using a mutant K-RAS transgenic mouse model, Schulz et al. (2014) demonstrated that an HFD could promote intestinal carcinogenesis by altering the microbial community. These studies thus indicated that a tumor-favorable microenvironment is vital for tumor progression and development. A chronic inflammatory microenvironment supports lung cancer cell development and metastasis ability (O'Callaghan et al., 2010; Tan et al., 2021). In addition, chronic inflammation has been reported to be associated with obesity (Ellulu et al., 2017; Rodríguez-Hernández et al., 2013). Although evidence regarding a relationship between obesity, lung cancer and chronic inflammation is accumulating, many aspects including the underlying molecular mechanisms have yet to be clarified.

We here focused on mutant epidermal growth factor receptor (EGFR)-driven lung cancer, which is common in non-smokers or light smokers in East Asia. A genetically engineered inducible EGFR L858R transgenic mouse model, identified as a model of human lung cancer development that can be used to assess the effectiveness of drugs (Taguchi et al., 2011; Politi et al., 2010), was used for investigating lung cancer progression in mice with or without an HFD. According to our results from cytokine and adipokine arrays, C-reactive protein (CRP) is a highly correlative factor in HFD groups. CRP is not only synthesized primarily in the liver but also in other cells, such as macrophages, lymphocytes, smooth muscle cells and adipocytes. It is well recognized as a biomarker of acute inflammation that can increase up to 1000-fold during infectious or inflammatory processes (Pepys and Hirschfield, 2003; Sproston and Ashworth, 2018). CRP has recently been demonstrated to be related to chronic inflammation and diseases (Luan and Yao, 2018). In addition, many correlation studies on CRP and lung cancer have demonstrated that elevated CRP levels are associated with increased lung cancer risk (Chaturvedi et al., 2010; Ji et al., 2022), and it has been suggested that CRP is a pre-diagnostic marker for lung cancer (McDonald et al., 2019; Chaturvedi et al., 2010; Xiao et al., 2019; Muller et al., 2019). However, few observational studies have shown that CRP affects lung cancer progression. Based on our results, we identified CRP as a valuable HFD-induced modulator that can facilitate lung cancer progression, and suggest that it could serve as a potential target for lung cancer monitoring and therapy.

## RESULTS

### HFD promotes lung cancer development in doxycycline-inducible lung cancer mice

**Fig. 1.**
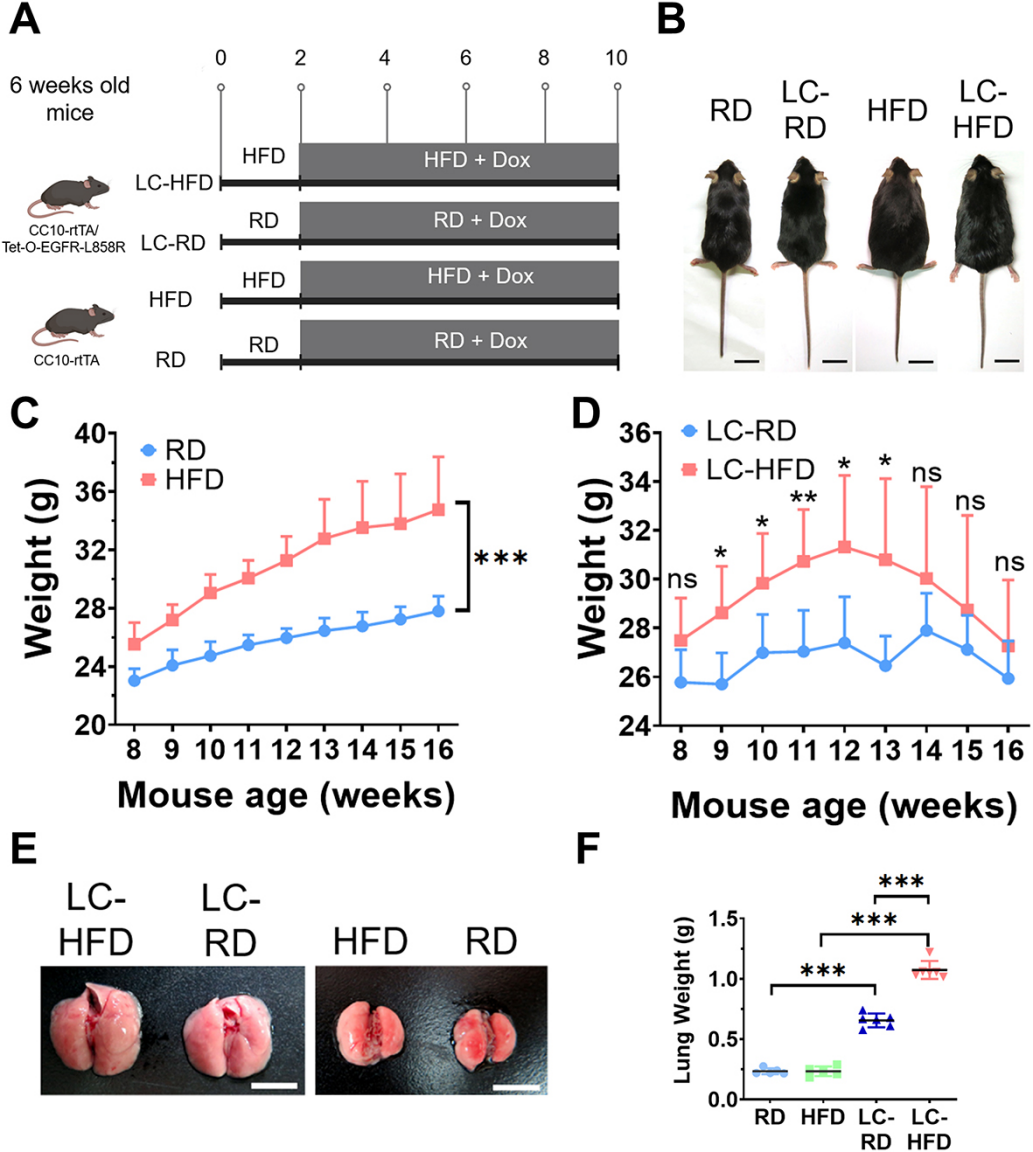
**HFD promotes lung cancer progression in the mutant EGFR-driven transgenic mouse model.** (A) Schematic of the experimental animal design. Six-week-old adult transgenic mice were randomly divided into four groups: LC-HFD, LC-RD, HFD and RD. LC-HFD and LC-RD mice had double transgenes (CC10-rtTA/Tet-O-EGFRL858R). They were fed an HFD or RD for 2 weeks and then switched to HFD+Dox or RD+Dox, respectively, for 8 weeks. HFD and RD mice had a single transgene (CC10-rtTA). They were fed an HFD or RD for 2 weeks and then switched to HFD+Dox or RD+Dox, respectively, for 8 weeks. Mutant EGFR-driven lung cancer was induced by Dox. (B) Representative photographs of mice at the end of the experiments. Scale bars: 2 cm. (C) Growth curve of mice in the HFD and RD groups (*n*=6 for each group). (D) Growth curve of mice in the LC-HFD and LC-RD groups (*n*=6 for each group). (E) Representative photographs of mouse lungs from each group. Scale bars: 1 cm. (F) Lung weight measurements of mice from each group at the end of the experiments. Results are shown as mean±s.d. Two-way ANOVA was used for statistical analysis in C; unpaired two-tailed Student's *t*-test was used for statistical analysis in D and F. ns, not significant; *P<0.05; **P<0.01; ***P<0.001. Dox, doxycycline; HFD, high-fat diet; LC, lung cancer; RD, regular diet.

To investigate the effects of HFD on lung cancer progression, adult male 6-week-old inducible transgenic mice were randomly separated into four groups (*n*=6 each): experimental groups, HFD lung cancer (LC-HFD) and regular diet (RD) lung cancer (LC-RD); and control groups, HFD and RD (Fig. 1A). The LC-HFD group consisted of transgenic mice (CC10-rtTA/tet-O-EGFR-L858R) treated with an HFD for 2 weeks, followed by induction with an HFD containing doxycycline (Dox) for 8 weeks. The LC-RD group consisted of transgenic mice (CC10-rtTA/tet-O-EGFR-L858R) treated with an RD for 2 weeks, followed by induction with an RD containing Dox for 8 weeks. As controls, the HFD and RD groups consisted of transgenic mice (CC10-rtTA) treated with HFD and RD for the first 2 weeks, then fed HFD+Dox and RD+Dox, respectively, for another 8 weeks. At the end of the experiment, lungs of mice from all groups were collected for histological analysis, immunohistochemical (IHC) analysis and RNA extraction. Their plasma samples were also collected for further analysis. To evaluate the effects of the HFD, the macroscopic appearance of the lungs and body weight were observed and recorded during the experiments. Compared with the RD group, the HFD group exhibited a significant increase in body weight (Fig. 1B,C). Compared with the LC-RD group, the body weight of the LC-HFD group increased significantly at the early induction stage but decreased notably at the late induction stage, presumably due to tumor overgrowth (Fig. 1D). Images obtained after dissection revealed that the mouse lung was enlarged in the LC-HFD and LC-RD groups compared with the control groups owing to lung cancer induction (Fig. 1E). In the mice with lung cancer induction, those fed an HFD had significantly increased lung weight compared with that of those fed the RD (Fig. 1F). However, the lung weight of the HFD and RD groups did not exhibit statistically significant differences (Fig. 1F). These results demonstrate that HFD can exacerbate lung cancer development.

### HFD promotes mutant EGFR-driven lung tumorigenicity

**Fig. 2.**
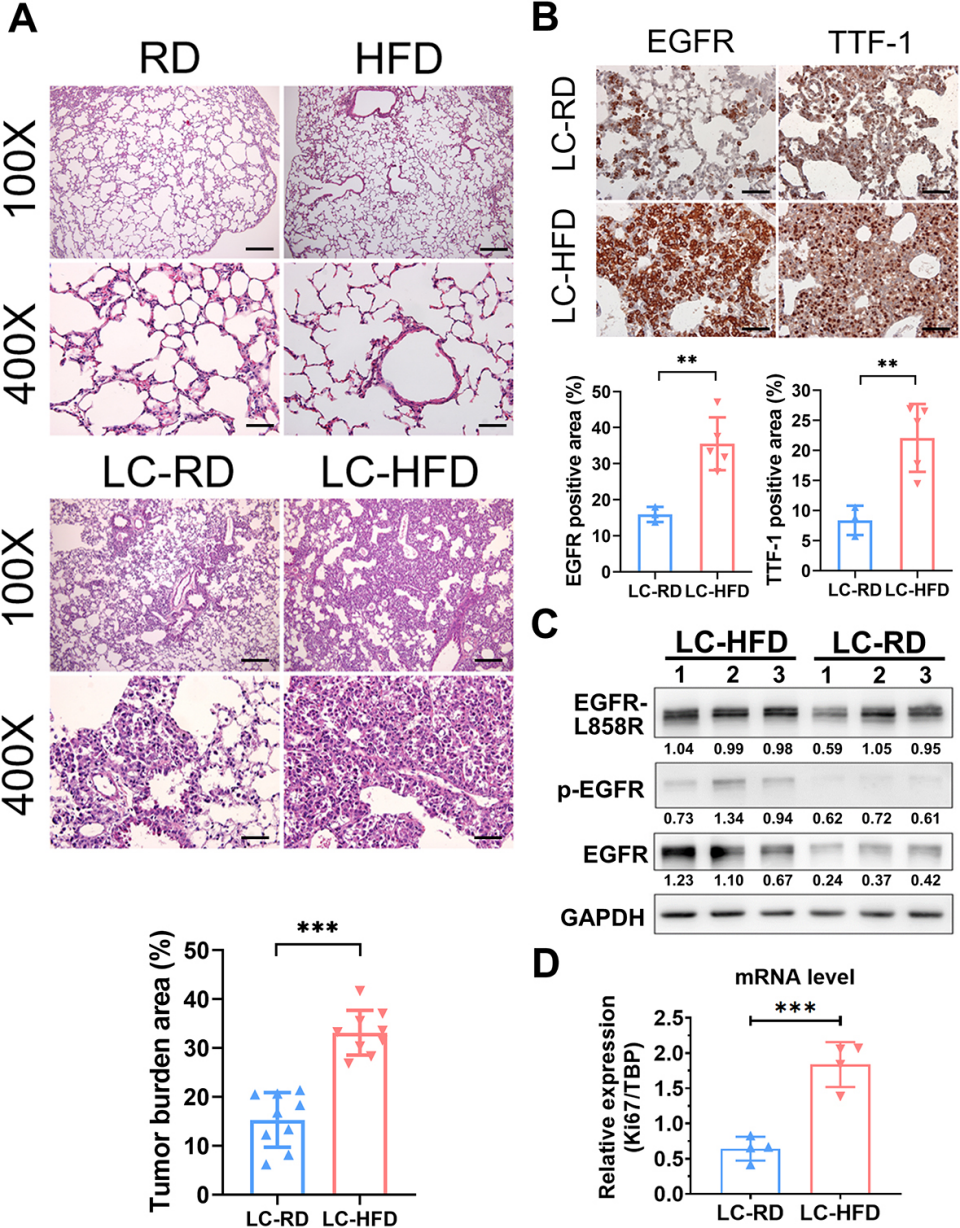
**Histopathological analysis and molecular characterization of lung tumor tissues in mice.** (A) Top panel: representative H&E staining of lungs from each group of mice at the end of the experiments. Scale bars: 100 μm (100×); 25 μm (400×). Bottom panel: quantification of tumor burden in LC-HFD and LC-RD mice (*n*=9 for each group). (B) Top panel: immunohistochemical analysis of EGFR and TTF-1 for evaluating tumor burden. Scale bars: 25 μm. Bottom panel: quantification of EGFR- and TTF-1-positive area in LC-HFD (*n*=5) and LC-RD (*n*=3) mice. (C) Western blot analysis of EGFR expression and activity. The quantified protein levels relative to GAPDH internal control are indicated below bands. (D) Quantification of *Ki67* mRNA levels for evaluating proliferative tumor cells in the LC-HFD and LC-RD groups (*n*=4 for each group). Results are shown as mean±s.d., and unpaired two-tailed Student's *t*-test was used for statistical analysis. **P<0.01; ***P<0.001.

To further investigate the tumor burden of each group, we performed histopathological analysis of the mouse lungs (Fig. 2). Hematoxylin and Eosin (H&E) staining indicated that the HFD could significantly increase the severity of lung tumor burden compared with the RD in mice with lung cancer induction (Fig. 2A). IHC analysis of EGFR and thyroid transcription factor-1 (TTF-1), a sensitive marker of primary lung adenocarcinomas (Reis-Filho et al., 2000), showed significant enhancement in LC-HFD mice compared with LC-RD mice, which confirmed the H&E results (Fig. 2B). To test whether the tumor burden was a consequence of EGFR L858R expression, western blotting was performed. Increased protein levels of total and phosphorylated (p-)EGFR were found in the LC-HFD group compared with those in the LC-RD group using antibodies against EGFR L858R and p-EGFR, respectively (Fig. 2C). Moreover, mRNA expression of the proliferation marker, *Ki67* (*Mki67*), was identified as being more upregulated in the LC-HFD lungs than in the LC-RD lungs by quantitative PCR (qPCR) analysis (Fig. 2D). Thus, an HFD can increase lung tumor burden and progression in the mutant EGFR-driven mouse model.

### Immune response and cytokine stimulation signaling are enriched in LC-HFD mice

**Fig. 3.**
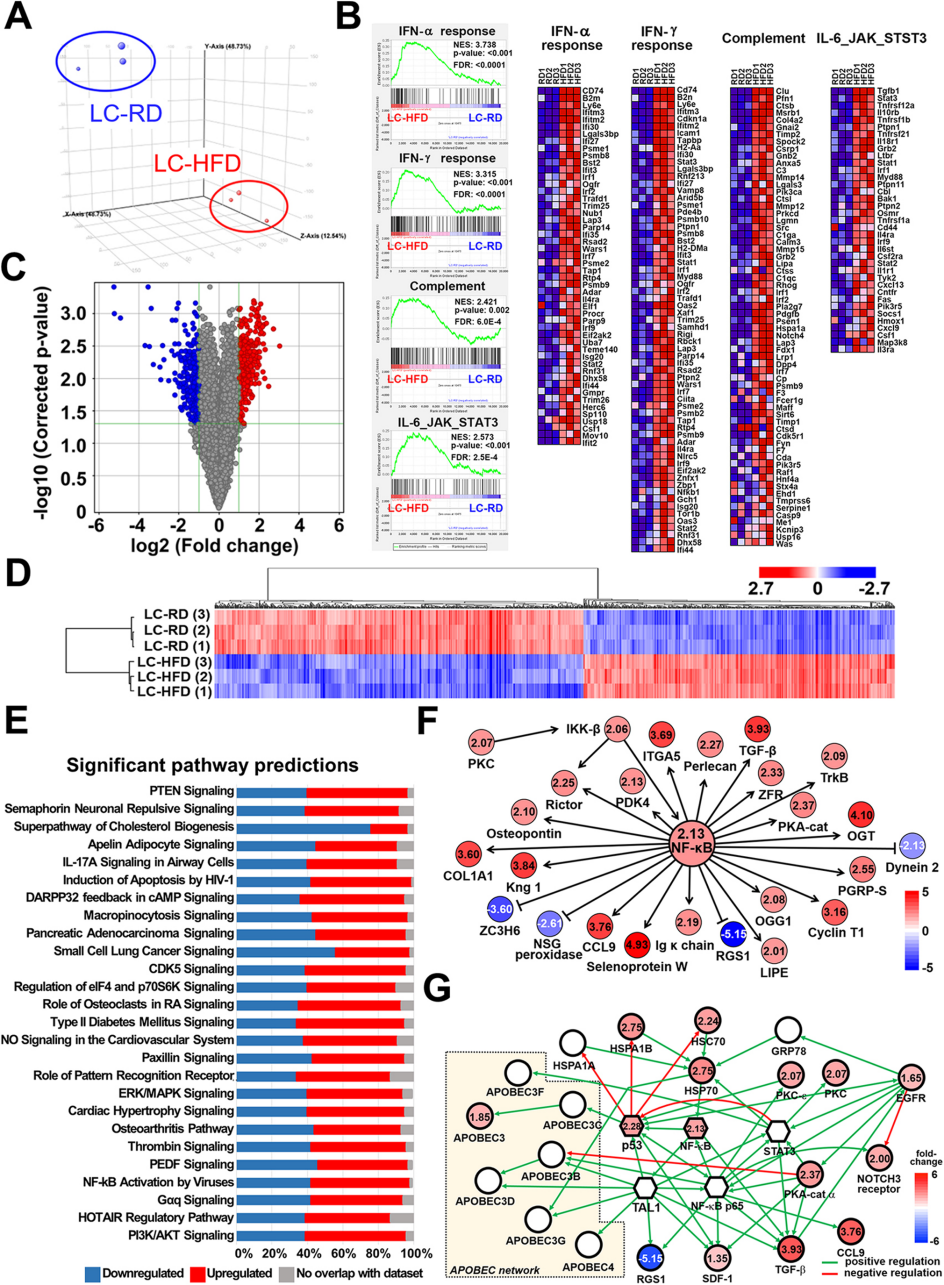
**Whole-genome transcriptomic analysis and regulatory network construction for HFD-induced lung cancer progression.** Total RNAs from three independent mouse lungs of the LC-HFD and LC-RD groups were reverse transcribed to cDNA and subjected to cDNA expression microarray experiment and analysis. (A) Principal component analysis of the global gene expression data from the LC-HFD and LC-RD groups (*n*=3 for each group). (B) Left panel: gene set enrichment analysis of whole-genome transcriptomic profiling using the cDNA expression microarray. Highly significantly enriched gene sets in LC-HFD group are shown. The green curve represents the density of the genes identified in the microarray. The FDR was calculated by comparing the actual data with the output of 100 Monte-Carlo simulations. The NES describes the density of modified genes in the dataset assuming random distribution. Right panel: heat maps of significantly enriched genes in each hallmark in the LC-HFC group, including IFN-α response, IFN-γ response, complement and IL-6/JAK/STAT3 signaling. (C) Volcano plot of differentially expressed genes (LC-HFD versus LC-RD). Blue dots represent genes that were significantly downregulated (*n*=221); red dots represent genes that were significantly upregulated (greater than twofold; *P*<0.05, unpaired two-tailed Student's *t*-test) (*n*=298). (D) Unsupervised hierarchical clustering analysis of 519 genes with significant changes. (E) Significantly enriched canonical pathways were predicted using IPA software. The bar on the right side of a pathway's name represents the percentage of related genes that were downregulated (blue), upregulated (red) and not mapped (gray). (F,G) Regulatory networks by NF-κB core transcriptional regulation (F), and potential modulations by EGFR and NF-κB signaling (G) for HFD-enhanced lung cancer. The network was constructed based on Metacore™ software analysis. Red and blue colors represent upregulated and downregulated genes, respectively, with the fold changes presented in numbers according to cDNA microarray results. FDR, false discovery rate.; NES, normalized enrichment score.

To elucidate the potential biological process and underlying mechanisms through which HFD exacerbates lung cancer, a cDNA expression array was used and analyzed, comparing the LC-HFD and LC-RD groups. Based on the results from the transcriptomic profiling of LC-HFD mice (Fig. 3), distinct clusters corresponding to the LC-HFD or LC-RD groups were identified in the principal component analysis (Fig. 3A). To understand the potential biological changes, the complete gene expression dataset was subjected to gene set enrichment analysis (GSEA) (Subramanian et al., 2005). Several critical gene set hallmarks and their corresponding gene expressions, including IFN-α response, IFN-γ response, complement and IL-6/JAK/STAT3 signaling, were positively enriched in the LC-HFD mice, suggesting that HFD helps cancer cells create a microenvironment related to immune and cytokine signaling and thus promotes cancer cell growth (Fig. 3B). Analysis of differentially expressed genes revealed that 519 genes (298 upregulated and 221 downregulated) (LC-HFD versus LC-RD, Fig. 3C; Table S1) exhibited a significant change (greater than twofold, *P*<0.05, unpaired two-tailed Student's *t*-test, based on false discovery rate). Genes with significant fold changes were used for the cluster analysis. Unsupervised hierarchical clustering of gene expression values revealed segregation between the LC-HFD and LC-RD groups (Fig. 3D). For the prediction of potential signaling pathways, genes with fold changes of more or less than two were analyzed using Ingenuity Pathway Analysis (IPA) software. Twenty-six significant canonical pathways were identified based on the criteria of *P*<0.05 and z-score >2 or <−2 (Fig. 3E; Table S2). Among these pathways, the presence of PI3K/AKT signaling, HOTAIR regulatory pathway, Gαq signaling, ERK/MAPK signaling, regulation of eIF4 and p70S6K signaling, CDK5 signaling, small cell lung cancer signaling and PTEN signaling implied that an HFD promoted lung cancer development by regulating cell cycle and proliferation signaling pathways. In addition, three immune response-related pathways were identified, including NF-κB activation by viruses, IL-17A signaling in airway cells and PEDF (SERPINF1) signaling, which suggested that a chronic inflammatory microenvironment was formed owing to immune modulation. Because macrophages are the main component in most cases of obesity-induced chronic inflammation (Kiran et al., 2021), we performed IHC analysis using antibodies against CD68 and F4/80 to demonstrate that macrophages were highly associated with HFD-enhanced lung cancer progression (Fig. S1). To construct the HFD induction-modulated molecular regulatory network, a transcriptional network of the upregulated and downregulated genes was built using Metacore™ software with the transcription regulation algorithm's default settings (Fig. 3F). Based on the prediction results, the constructed network indicated that NF-κB was a core transcription factor, and that several immune- or cytokine response-related downstream genes were regulated. To further uncover the underlying molecular regulatory network involved in HFD-induced lung cancer progression, we tried to construct the signaling networks based on NF-κB signaling, EGFR and the significantly altered genes in our cDNA array (Fig. 3G). HFD might promote NF-κB signaling through EGFR-mediated heat shock proteins or protein kinase A molecules. Furthermore, the APOBEC network, which can promote cancer progression or metastasis or drug resistance under NF-κB signaling (Isozaki et al., 2023; Maruyama et al., 2016), may be downstream modulated by NF-κB/p53 signaling (Fig. 3G). Although only APOBEC3 had slight upregulation, whether the APOBEC network can mediate HFD-induced lung cancer progression requires further exploration. These results suggest that HFD increases lung tumor burden, possibly owing to cytokine and immune microenvironment modulation.

### CRP is a central cytokine in the immune microenvironment promoting tumor progression

**Fig. 4.**
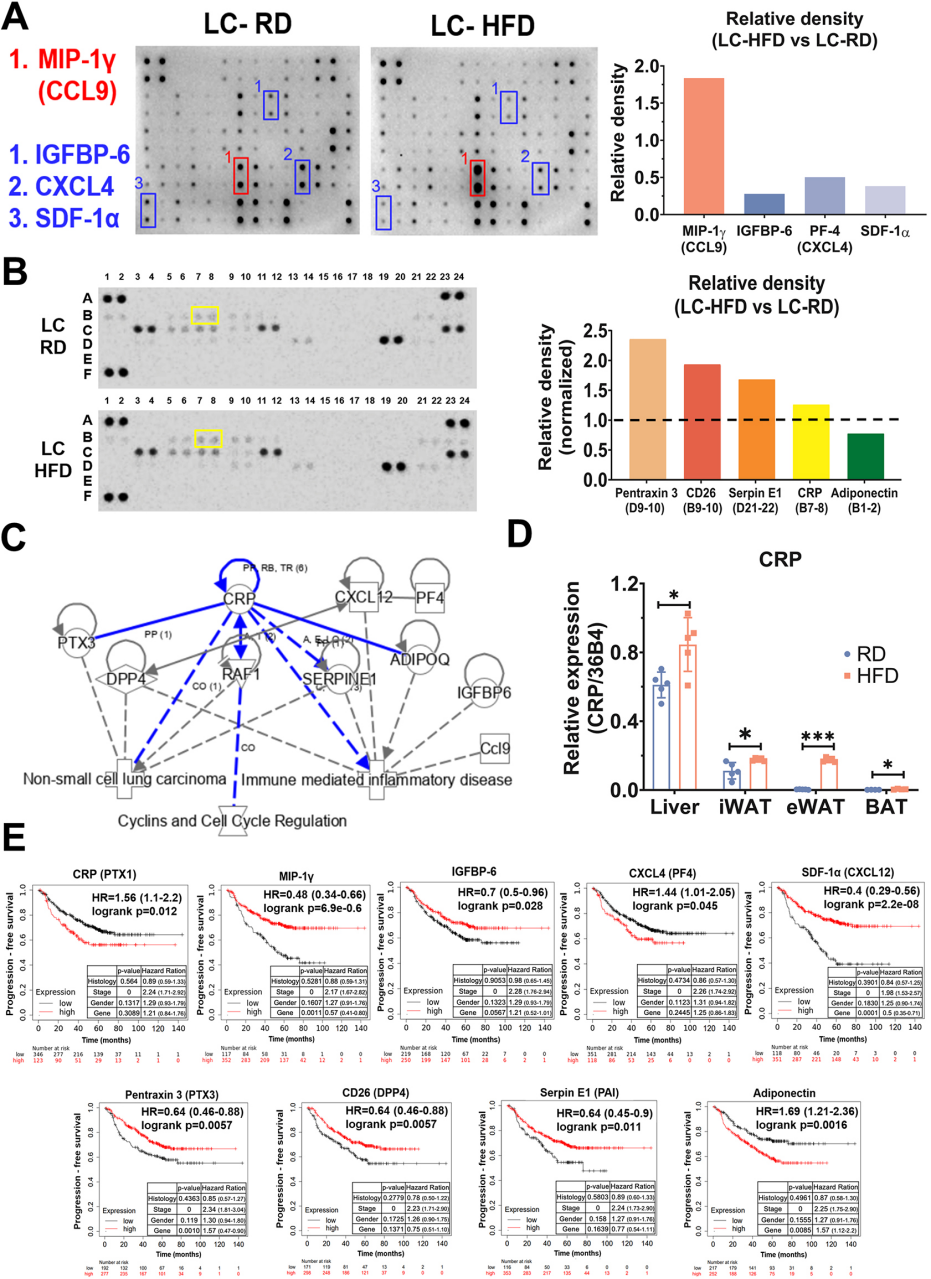
**Identification of cytokines and adipokines potentially contributing to HFD-induced lung cancer progression.** Mouse lung tissue was used to profile a total of 62 cytokines, and the serum was used to profile a total of 38 adipokines through antibody arrays. IPA software was used to further analyze the results on regulatory network prediction and the correlation of clinical outcomes through KM plotter analysis. (A) Left panel: a cytokine antibody array was prepared using lung cancer tissues from LC-HFD and LC-RD mice. The red and blue boxes represent upregulated and downregulated cytokines, respectively, in the LC-HFD compared with LC-RD group. Right panel: quantification of cytokines using ImageJ software. (B) Left panel: the levels of 38 different adipokines in mouse sera. The yellow boxes represent CRP upregulated in the LC-HFD group compared with that in the LC-RD group. Right panel: quantification of adipokines using ImageJ software. (C) Establishing the potential regulatory network underlying cytokine and adipokine modulation in HFD-treated mice with lung cancer using IPA software. (D) Quantitative real-time PCR analysis of *Crp* mRNA expression levels in the liver and adipose tissues. (E) Prognostic prediction power for correlation between progression-free survival and the cytokines and adipokines mentioned in A and B. The survival data were analyzed by KM plotter analysis combined with multivariate Cox proportional hazards regression analysis. Patients were grouped according to auto-selection of the best cut-off. *P<0.05; ***P<0.001. BAT, brown adipose tissue; CRP, C-reactive protein; eWAT, epididymal white adipose tissue; HR, hazard ratio; iWAT, inguinal white adipose tissue.

Focusing on HFD-induced obesity-related inflammation, adipokines secreted by adipose tissues impacted inflammatory response modulation (Kawai et al., 2021). Also, cytokines were critical molecules to mediate immune response in the tumor microenvironment (Lan et al., 2021). We hypothesized that adipokines secreted in response to an HFD are distributed to lung cancer tissue through the circulation and promote tumor progression. To connect HFD-induced signal networks in lung cancer tissue, we profiled cytokines and adipokines, representing systematic immune-related secretions and obesity-related productions, respectively. First, we performed a mouse cytokine antibody array for the profiling of 62 cytokines, chemokines, growth factors and soluble receptors using lung tissues from each mouse group (Fig. 4A, left panel). The results revealed that MIP-1γ (CCL9) was most significantly upregulated in the LC-HFD group compared with the LC-RD group (Fig. 4A, left panel, red box). By contrast, three factors, namely IGFBP-6, CXCL4 (PF4) and SDF-1α (CXCL12), were considerably downregulated (Fig. 4A, left panel, blue box). Second, because HFD is known to alter adipokine secretion in the body (Zorena et al., 2020), we performed adipokine profiling using the peripheral blood of LC-HFD and LC-RD mice to identify candidates contributing to lung cancer (Fig. 4B, left panel). The results revealed that, among 38 adipokines, pentraxin 3 (PTX3), CD26 (DPP4), serpin E1 (SERPINE1) and CRP were upregulated, whereas adiponectin (ADIPOQ) was downregulated 1.25-fold, in the LC-HFD mice (Fig. 4B, right panel). A comprehensive analysis of the cytokine and adipokine arrays revealed immune microenvironment modulation in the LC-HFD group; this result was consistent with the enrichment analysis results (Fig. 3B). Moreover, the profiling suggested that HFD-generated obesity, a state accompanying chronic inflammation, can promote lung cancer development. To further expand our understanding of the regulatory networks involved in lung cancer, cell cycle and inflammation, a network of the mentioned cytokines and adipokines with significant changes in array profiling was constructed using IPA software (Fig. 4C). Among the identified factors with different expression, CRP was associated with obesity, chronic inflammation and lung cancer risk. Furthermore, CRP was found to act as a central factor connecting other cytokines and adipokines that contributed to lung cancer progression, suggesting that it might act as a bridge linking these aspects. We further addressed the potential cellular origin of CRP for lung cancer progression by performing qPCR analysis to evaluate mRNA expression levels in the liver and adipose tissues (Fig. 4D). *Crp* expression was increased in all tissues, especially in the liver and epididymal white adipose tissue (eWAT), suggesting that it contributes to tumor–immune interaction and lung cancer progression through the circulation. To further validate the finding by leveraging public datasets, the Kaplan–Meier (KM) plotter was used to analyze the correlation between the transcriptional expression of the secretory factors and clinical outcomes in lung cancer patients (Fig. 4E). Following multivariate adjustments made for histology, stage and sex, the mRNA levels of all cytokines and adipokines were found to be independent factors significantly correlated with progression-free survival and overall survival (Fig. 4E; Fig. S2). However, only *Crp* and *Sdf1a* mRNA levels were correlated with the effects of the HFD, suggesting that both factors promote lung cancer progression under an HFD. These results thus indicate the involvement of CRP in the relationship among obesity, immunity and lung cancer progression.

### CRP promotes the proliferative, migratory and invasive ability of lung cancer cells

**Fig. 5.**
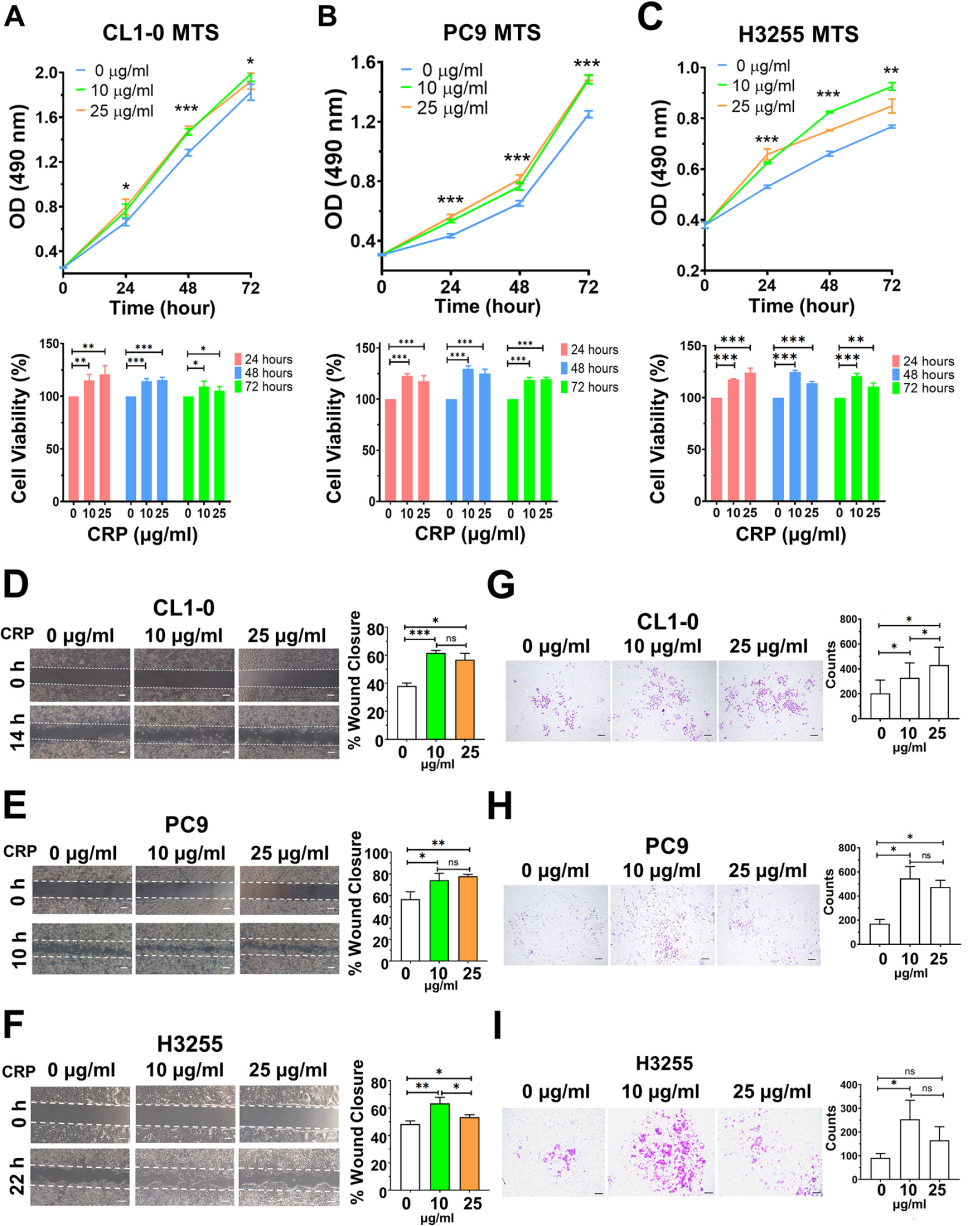
**CRP enhances lung cancer cell proliferative ability and invasiveness.** (A-C) Evaluation of cell proliferation through MTS assay of CRP-treated CL1-0 (A), PC9 (B) and H3255 (C) lung adenocarcinoma cells (top panel), and quantification of relative proliferative abilities (bottom panel). (D-F) Evaluation of cell migration through wound-healing assays of CRP-treated CL1-0 (D), PC9 (E) and H3255 (F) lung adenocarcinoma cells (left panel), and quantification of relative migratory abilities (bottom panel). Dashed lines in D-F indicate the initial cell boundary. (G-I) Evaluation of cell invasiveness through Matrigel transwell assays of CRP-treated CL1-0 (G), PC9 (H) and H3255 (I) lung adenocarcinoma cells (left panel), and quantification of invaded cells (right panel). Scale bars: 20 μm. Results are shown as mean±s.d., and unpaired two-tailed Student's *t*-test was used for statistical analysis. ns, not significant; *P<0.05; **P<0.01; ***P<0.001. OD, optical density.

**Fig. 6.**
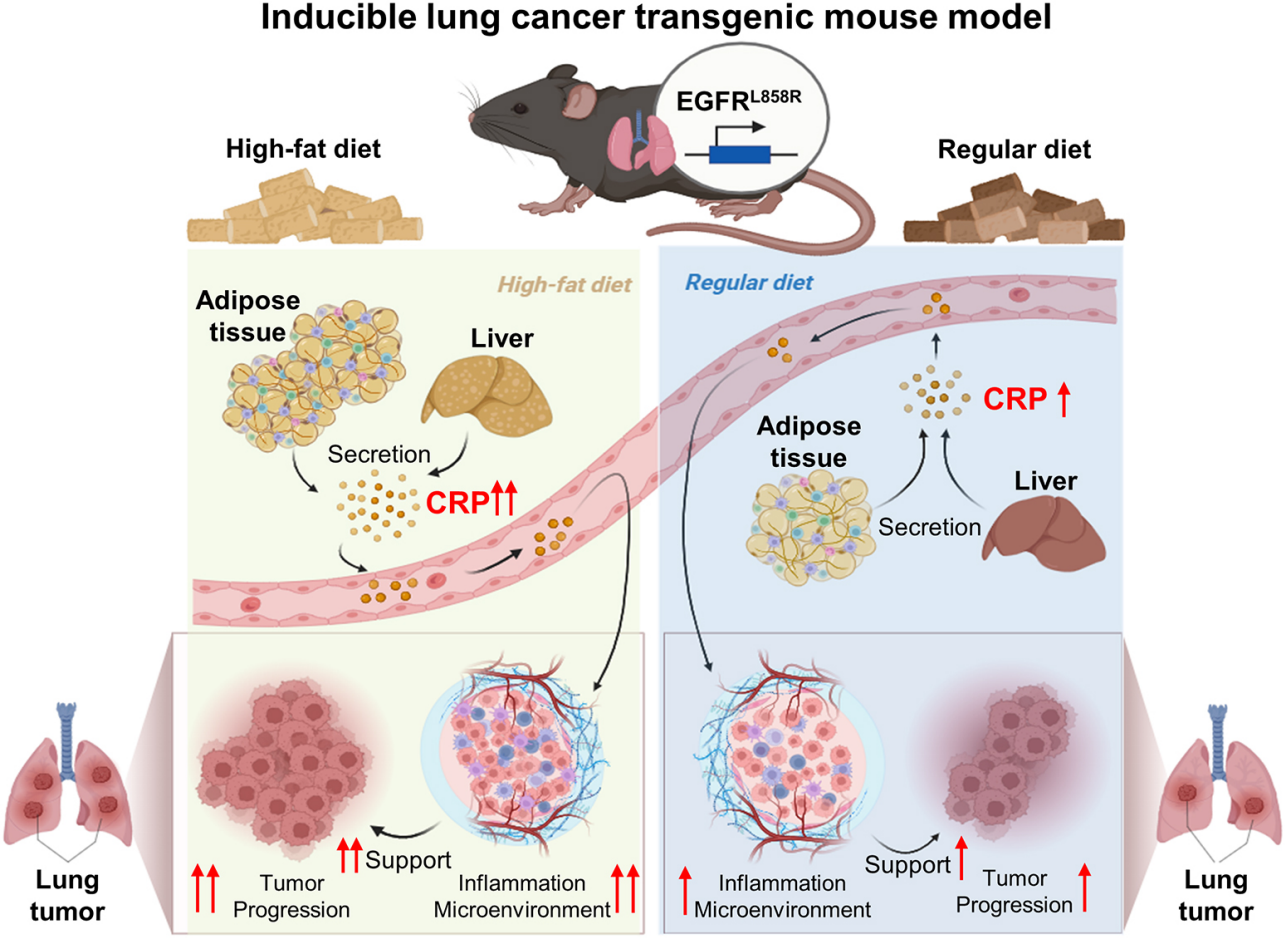
**HFD-induced CRP secretion and lung cancer progression.** HFD increases CRP secretion from the liver and adipose tissue in mice with lung cancer. Circulating CRP modulates immune responses and induces inflammation in the tumor microenvironment. CRP stimulates tumor cell proliferation, promoting lung cancer progression.

To test whether CRP can enhance the characteristics of tumorigenicity in lung cancer cells with or without EGFR mutations, we conducted proliferation, migration and metastasis assays using CL1-0 (wild-type EGFR), PC9 (EGFR exon19 deletion) and H3255 (EGFR L858R) lung adenocarcinoma cell lines treated with different CRP dosages (Fig. 5). Based on previous studies, minor-elevation (1 μg/ml) and moderate-elevation (2.5 μg/ml) dosages of CRP were used to represent the clinical features of a chronic inflammation process in obesity and in chronic diseases (Gewurz et al., 1982; Soinio et al., 2006). Exogenous CRP in a culture medium significantly elevated cell proliferation in all cell lines (Fig. 5A-C). CRP significantly increased cell proliferation in a dose-dependent manner, as shown by MTS assay. We next tested the effects of CRP on cell migration and invasion. In wound-healing assays, CRP enhanced the migratory ability of all cell lines (Fig. 5D-F). Furthermore, transwell invasion assays were performed in all cell lines to evaluate the impact of CRP on cancer cell invasiveness, and exogenous CRP could significantly promote cell invasiveness (Fig. 5G-I). In conclusion, HFD-induced CRP promotes lung cancer progression by modulating the immune response and enhancing cancer cell tumorigenicity (Fig. 6).

## DISCUSSION

Obesity has become a critical health problem worldwide and represents a substantial risk for several chronic diseases and cancer (Pi-Sunyer, 2009). HFD contributes to excessive adipose tissue accumulation that can harm multiple tissues through different mechanisms (Sun et al., 2020; Jiang et al., 2020; Gao et al., 2015). Many observational studies have reported a link between obesity and cancer risk (Quail and Dannenberg, 2019; Pati et al., 2023), but establishing the causal relationship is challenging. Ringel et al. (2020) reported that HFD might compromise anti-tumor immunity by interfering with metabolism in the tumor microenvironment and that the impact of HFD on tumor cell growth may be correlated with its immunogenic properties. In this study, we used a transgenic mouse model to investigate the effects of HFD on cancer progression. This model enabled us to realistically study the tumor microenvironment due to the occurrence of endogenous and spontaneous tumor formation. Based on the results of whole-genome transcriptomic analysis (Fig. 3), especially GSEA, IPA and Metacore™ analyses, we propose that an HFD triggers the induction of chronic inflammation, thereby facilitating lung cancer development. Hill et al. (2023) recently showed that air pollution promotes macrophage influx into the lungs and IL-1β release, driving a progenitor-like state of EGFR mutant alveolar type II epithelial cells into tumorigenesis (Hill et al., 2023). This demonstrated that systemic immunity was linked to cancer progression, as reported in previous studies (Dunn et al., 2002; Chew et al., 2012). These results imply that the chronic inflammation microenvironment can potentially promote cancer progression. In addition to mutant EGFR, mutant KRAS (G12C or G12D), more frequently found in smokers, might be involved in HFD-induced lung cancer progression. Previous studies had mixed conclusions regarding the impact of diet on mutant KRAS-driven cancers owing to complicated downstream signaling and other confounding factors (Ramadori et al., 2015; Norris et al., 2015; Chung et al., 2020; Lauby-Secretan et al., 2016). These findings also reflected the limited representativeness of the mouse model employed in this study, which only captures a portion of the HFD-induced lung cancer progression. To comprehensively understand the impact of an HFD on all types of lung cancer, further investigations should encompass other lung cancer models.

Obesity is well known to be associated with a greater abundance of immune cells – such as macrophages, neutrophils, T cells, B cells and mast cells – around tissues (Nishimura et al., 2009; Osborn and Olefsky, 2012; Winer et al., 2011). Macrophages were the main component in most obesity-induced chronic inflammation, leading to related metabolic disorders (Kiran et al., 2021). In people with HFD-induced obesity, massive accumulation of macrophages accompanied by a shift in the ratio of M2/M1 macrophages, compared with those in controls, has been documented (Kanneganti and Dixit, 2012; Martinez et al., 2009). Furthermore, HFD-induced obesity has been reported to be associated with chronic inflammation within adipose tissue caused by accumulation of CD4^+^ and CD8^+^ T cells (Anderson et al., 2010; Winer and Winer, 2012). Also, Th17 cells may contribute to obesity-induced inflammation, based on a previous study that revealed that Th17 cells and IL-17 were positively correlated with BMI (Schindler et al., 2017). These findings implied that the immune cell composition of the chronic inflammatory microenvironment is altered after an HFD. Single-cell RNA-sequencing technology facilitated a comprehensive profile of immune cell composition after HFD challenge in mouse liver or adipose tissue (Su et al., 2021; Yang et al., 2022). Although lung tissue was less discussed, a study indicated that CD68^+^ macrophages were upregulated in the lungs of mice after an HFD, which supported our results (Fig. S1) (Qi et al., 2020). It has also been reported that unbalanced pro- and anti-inflammatory cytokines promote obesity-associated disease progression (Chawla et al., 2011).

Our results demonstrated that an HFD increases CRP expression, promoting an inflammatory environment and generating immune responses that benefit tumor growth (Figs 3-5). Moreover, regulatory network analysis indicated that CRP might be a central factor in modulating sequential cascades, thereby contributing to lung cancer progression (Fig. 4C). Elevated CRP levels were strongly associated with lung cancer risk as a chronic inflammation marker and, subsequently, as a lung carcinogenesis biomarker (Chaturvedi et al., 2010; Gaur et al., 2019; Ji et al., 2022). Despite evidence supporting the application of CRP measurements for lung cancer risk stratification (Hart et al., 2020; Li et al., 2014), the exact etiological role of CRP in lung cancer requires further clarification. CRP activates the NF-κB signaling pathway associated with inflammation activation (Ji et al., 2022). Additionally, our regulatory network analysis demonstrated that NF-κB functions as a central transcription factor modulating multiple downstream molecules (Fig. 3F). These results suggest that an HFD contributes to establishing an inflammatory microenvironment for facilitating lung tumor growth through the secretory CRP/NF-κB axis.

In addition to CRP, significant changes in cytokines such as MIP-1γ, SDF-1α and IGFBP-6 were observed in the LC-HFD lungs compared with the LC-RD lungs (Fig. 4). MIP-1γ, also known as CCL9, is often produced in macrophages and osteoclasts. It promotes cell survival by promoting NF-κB signaling and mediates T-cell migration (Okamatsu et al., 2004; Müller et al., 2003). SDF-1α can regulate tumor cell proliferation, motility, epithelial–mesenchymal transition and metastasis by binding to CXCR4 and activating downstream signaling (Marchesi et al., 2004; Otsuka and Bebb, 2008; Wang et al., 2021). IGFBP-6, an IGFBP family member that can increase the half-life of circulatory insulin-like growth factors (IGFs), is a serum biomarker associated with poor prognosis in lung cancer and immune cell regulation (Cai et al., 2020; Sueoka et al., 2000). Nevertheless, the mechanisms underlying the effects of MIP-1γ, SDF-1α and IGFBP-6 in lung cancer have not been clearly defined. The significant changes in these cytokines observed in our study might indicate that different cytokines regulate immune function by modulating the tumor microenvironment in lung cancer under HFD stimulation. However, the KM plotter outcome analysis presented some results conflicting with those obtained using our animal model (Fig. 4E; Fig. S2). This reflects the heterogeneity and complexity of the tumor microenvironment in HFD-induced lung cancer, which may differ slightly between humans and mice.

Diet-induced obesity-enhanced cancer progression has been documented (Kawai et al., 2021), and we here investigated the link between diet, adipokines and lung cancer progression. In addition to CRP, PTX3, CD26, serpin E1 and ADIPOQ were altered under HFD stimulation (Fig. 4). PTX3 can modulate the progression and chemoresistance of non-small cell lung cancer (NSCLC) by activating AKT/NF-κB signaling (Li et al., 2021). CD26 expression is higher in lung cancer tissues than in normal tissues, and it can become a target for suppressing lung cancer growth (Jang et al., 2015, 2019). Serpin E1 has also been considered a reliable biological and prognostic marker for various cancers (Arroyo-Solera et al., 2019; Chen et al., 2022; Kong et al., 2021). Furthermore, downregulation of ADIPOQ, an adipocyte-derived adipokine, enhances migratory and invasive abilities in NSCLC (Cui et al., 2018).

The relationship between obesity and lung cancer outcome remains controversial. Although a large cohort study concluded that being underweight or obese was associated with survival in NSCLC patients (Shepshelovich et al., 2019), Yu et al. (2018) reported that low BMI was correlated with high-risk populations for lung cancer (Yu et al., 2018). Although BMI is commonly used to assess obesity, there remain many confounding factors than can affect the outcome of lung cancer. CRP might be an objective signature for either outcome prediction or disease monitoring. In addition, a systemic analysis showed that high CRP expression could predict prognosis and correlate with higher mortality in lung cancer and other solid tumors (Shrotriya et al., 2015), and another study found that lung cancer survival was proportionally decreased with higher serum CRP levels (Bittoni et al., 2015). The present study has provided insight into the connection between HFD, CRP and lung cancer progression. A prospective clinical study should be conducted to verify the findings in the future.

## MATERIALS AND METHODS

### Mutant EGFR-driven lung cancer mouse model

Dox-inducible lung cancer mice, Tg(CC10-rtTA/tet-O-EGFR-L858R), carried double transgenes by breeding two transgenic mice, Tg(CC10-rtTA) and Tg(tet-O-EGFR-L858R). Tg(CC10-rtTA) mice from the Research Animal Resource Center of Memorial Sloan Kettering Cancer Center expressed the reverse tetracycline-controlled transactivator (rtTA) protein under the control of the CC10 promoter. It provided a ‘Tet-On’ tool that allowed the inducible expression of genes in the adult lung and respiratory epithelium. Tg(tet-O-EGFR-L858R) mice from the National Cancer Institute (NCI) Model of Human Cancers Consortium (MMHCC) carried mutant human *EGFR* cDNA with a T-to-G nucleotide substitution in exon 21 that replaced leucine with arginine at amino acid position 858 (L858R) under control by a tetracycline-responsive element. Two transgenic mouse lines were backcrossed to C57BL/6 genetic background for over ten generations before experiments.

### Animal experiments and HFD for lung cancer induction

All animal studies were carried out in the AAALAC-accredited animal center of National Taiwan University Medical College and approved by the Institutional Animal Care and Use Committee (IACUC) (No. 20140453). The induction of lung cancer was according to Regales et al. (2007). Customized diets used in this study included 625 ppm Dox-containing RD (TestDiet, 5TWN), 625 ppm Dox-containing HFD (TestDiet, 58Y1) and HFD (TestDiet, 5W5W). Each diet was replaced twice a week. For animal experiments, 6- to 8-week-old male mice were used.

### Histological analysis

H&E stains of lung sections were performed by National Taiwan University Laboratory Animal Center according to standard procedures. Briefly, the tissue section was deparaffinized by three times xylene treatment and serial downward-concentrated alcohol (100% to 50%) rehydroxylation procedures. After a 10 min wash, slides were stained with Hematoxylin (Sigma-Aldrich, GHS316) for 5 min, and then, following a water wash, stained with Eosin (Sigma-Aldrich, 17372871) for 1 min, before being mounted for microscope observation.

### IHC analysis

Serial 4 μm sections from the paraffin-embedded lung were mounted on silicon-coated slides, deparaffinized by xylene, and rehydrated through graded ethanol to water. After treatment with 3% H_2_O_2_, primary antibodies were incubated for 20 h overnight at 4°C. Immunostaining was performed by a Liquid DAB+ Substrate Chromogen System (DAKO, K3467) for 15 min and counterstain with Hematoxylin (Sigma-Aldrich, GHS316) for 10 s. Antibodies, including anti-EGFR (Cell Signaling Technology, 4267), anti-TTF1 (LSBio, LS-C154673), anti-CD68 (Abcam, ab125212), anti-F4/80 (Cell Signaling Technology, 70076) and IgG (Abcam, ab172730), were 1:100 diluted for IHC analysis.

### Immunoblotting

Lung tissues were lysed in M-PER or T-PER Tissue Protein Extraction Reagent (Thermo Fisher Scientific, 78510) containing an additional 1× PhosStop (Roche, 04906837001) phosphatase inhibitor and 1× protease inhibitor cocktail (Sigma-Aldrich, S8830), and protein was extracted according to the manufacturer's protocol. Samples were supplemented with 0.25 volumes of 4× sample buffer containing 2-mercaptoethanol. Samples were run on 10% SDS-PAGE gels, transferred to PVDF membranes, and blotted with anti-EGFR L858R (1:1000, Cell Signaling Technology, 3197), anti-EGFR (1:1000, Cell Signaling Technology, 4267), anti-p-EGFR (1:1000, Cell Signaling Technology, 2237) or anti-GAPDH (1:5000, Proteintech, 60004-1-Ig) antibodies. ImageJ software was used to semi-quantify the western blotting results.

### Real-time quantitative reverse transcription PCR

RNA was isolated from each mouse lung tissue and extracted by TRI reagent (Sigma-Aldrich, T9424-100ML), followed by a chloroform–isopropanol system. Reverse transcription was performed with Superscript III First-Strand Synthesis SuperMix (Invitrogen, 18080400). For qPCR reactions, SYBR Green PCR Master Mix was mixed with 40 ng cDNA and appropriate primers. The relative mRNA expression level of the target gene was determined as −ΔCT=−(CT_target_−CT_TBP_). The target/*Tbp* mRNA ratio was calculated as 2^−ΔCT^. Primer sequences were as follows: *Ki67* forward primer, 5′-AATCCAACTCAAGTAAACGGGG-3′; *Ki67* reverse primer, 5′-TTGGCTTGCTTCCATCCTCA-3′; *Tbp* forward primer, 5′-GCAGTGCCCAGCATCACTAT-3′; *Tbp* reverse primer, 5′-GCCCTGAGCATAAGGTGGAA-3′; *Crp* forward primer, 5′-TTCCTGAGGCTCCAACACAC-3′; *Crp* reverse primer, 5′-GCAGACTTTTCCGCACCTTG-3′; 36B4 (*Rplp0*) forward primer, 5′-AGATTCGGGATATGCTGTTGG-3′; 36B4 reverse primer, 5′-AAAGCCTGGAAGAAGGAGGTC-3′.

### cDNA array and IPA pathway analysis

Total RNA from the lungs was reverse transcribed to cDNA. The cDNA was purified and transcribed to biotinylated RNA, and then fragmented and hybridized to the Mouse Genome 430 2.0 GeneChip (Affymetrix). The chip was washed, stained and scanned with an Affymetrix GeneChip Scanner 3000 7G. Data on each lung were obtained from each mouse. The raw data-containing CEL file was further analyzed by Partek software. The selected genes for pathway analysis were chosen under twofold change-correlated *P*-value (<0.05), and IPA software was used for pathway analysis. Raw data were deposited in the Gene Expression Omnibus (GEO) and are available with the accession number GSE119649.

### GSEA

The gene expression data generated by RNA sequencing were analyzed using GSEA 4.0. Significantly enriched gene sets are shown under the following parameters: gene set database, mh.all.v2023.1.Mm.symbols.gmt; number of permutations, 1000; Collapse/Remap to gene symbols, Collapse; permutation type, gene_set; chip platform, Mouse_AFFY_Mouse430_MSigDB.v2023.1.Mm.chip; enrichment statistic, Classic; metric for ranking genes, Diff_of_Classes. The remaining parameters were maintained at their default values.

### Kaplan–Meier survival analysis

For survival analysis, the KM Plotter, an online database containing gene expression data and overall survival information on lung adenocarcinoma patients, was used (Györffy et al., 2013). Patients were grouped according to auto-selection of the best cut-off. *P*<0.05 was considered statistically significant. The multivariate Cox proportional hazards regression model to test the independent value of each gene among confounding factors including histology, stage and gender was performed according to the default parameter of KM Plotter.

### Cytokine array

Total protein from lung tissues was extracted by T-PER Tissue Protein Extraction Reagent (Thermo Fisher Scientific, 78510) and ultrasound sonication for 10 s three times (Ultraschallprozessor, UP50h). In each cytokine array reaction, 500 ng protein was used, according to the users' manual from the Mouse Cytokine Antibody Array C3 kit (Raybiotech, AAM-CYT-3-4).

### Adipokine array

A Proteome Profiler™ Array Mouse Adipokine Array Kit (R&D Systems, ARY013) was used to detect 38 different adipokines in mouse serums. Samples were centrifuged before mixing with a cocktail of biotinylated detection antibodies. All steps in the product datasheet were followed. Each sample's signal (relative density) was normalized to the positive control's signal. Detailed procedures were according to the commercial manual.

### Cell culture

CL1-0 (RRID, No. CVCL_3871), PC9 (RIKEN BRC, No. RCB0446) and H3255 (NCI-DTP, No. NCI-H3255) cells were cultured in Gibco RPMI1640 (Thermo Fisher Scientific) supplemented with 10% fetal bovine serum (FBS; Merck Millipore, TMS-013-BKR), 100 units/ml penicillin, 100 µg/ml streptomycin, 0.25 µg/ml amphotericin B (Thermo Fisher Scientific, 15240062) and 2 mM L-glutamine (GeneTeks, GT-SLG100) at 37°C and 5% CO_2_. Cells were free of mycoplasma, as detected by a Mycoplasma Detection Kit (BioSmart, BSMP101).

### MTS assay

The proliferation of CL1-0, PC9 and H3255 cells was detected by CellTiter 96^®^ AQueous Non-Radioactive Cell Proliferation Assay (Promega, G3580). Three thousand cells per well were initially seeded in 96-well plates. After 24 h incubation, fresh medium containing different concentrations of CRP (Sigma-Aldrich, AG723-M) was replaced in these seeding wells. Following another 24 h incubation, the medium was replaced with a solution containing MTS and PMS. Subsequently, the cells were incubated for 1 h in the incubator, and absorbance at 490 nm was recorded by an ELISA plate reader (Molecular Devices, SpectraMax iD3). Detailed procedures were according to the commercial manual.

### Invasion assay

BD Matrigel Matrix (BD Biosciences, 356234; 50 µl) was coated onto the upper chamber of each insert. For CL1-0 and PC9 cells, 1.2×10^4^ cells were trypsinized and seeded in the upper chamber with 100 µl culture medium containing 1.5% FBS, in the presence or absence of CRP. In the case of H3255 cells, 6×10^4^ cells were trypsinized and seeded in the upper chamber with 100 µl culture medium containing 1.5% FBS with or without CRP. The lower chamber was filled with 700 µl culture medium containing 10% FBS. Following a 48 h incubation at 37°C, the membranes were fixed using a 5% formaldehyde solution and subsequently stained with Crystal Violet (Sigma-Aldrich, C0775).

### Wound-healing assay

CL1-0 and PC9 cells were seeded in 12-well plates to confluence. Cell monolayers were scratched with a 200 µl tip, followed by medium removal, washing, and addition of fresh medium in the presence or absence of CRP. H3255 cells were seeded in a Culture-Insert 2 Well (ibidi, 80206) to confluence. Cells were cultured at 37°C for 14 h (CL1-0), 10 h (PC9) and 22 h (H3255), and photographed by inverted microscopy at 0 h and the final hour. Wound areas were measured using ImageJ software.

### Statistical analyses

Graphs were prepared using GraphPad Prism 8 software. Statistical analyses were conducted using unpaired two-tailed Student's *t*-test between two groups and reported as mean±s.d., except for data in Fig. 1C, which were analyzed using two-way ANOVA and reported as mean±s.d. *P*<0.05 was considered significant.

## Supplementary Material

10.1242/dmm.050360_sup1 Supplementary information

## First Person

This article has an associated First Person interview with the first author of the paper.
