# Supplementary material for: High-fat diet induces C-reactive protein secretion, promoting lung adenocarcinoma via immune microenvironment modulation
Source: Dis Model Mech. 2023 Nov 9;16(11):dmm050360. doi: 10.1242/dmm.050360 (PMC10651111; doi:10.1242/dmm.050360)
Supplement: Supplementary information [file dmm-16-050360-s1.pdf]

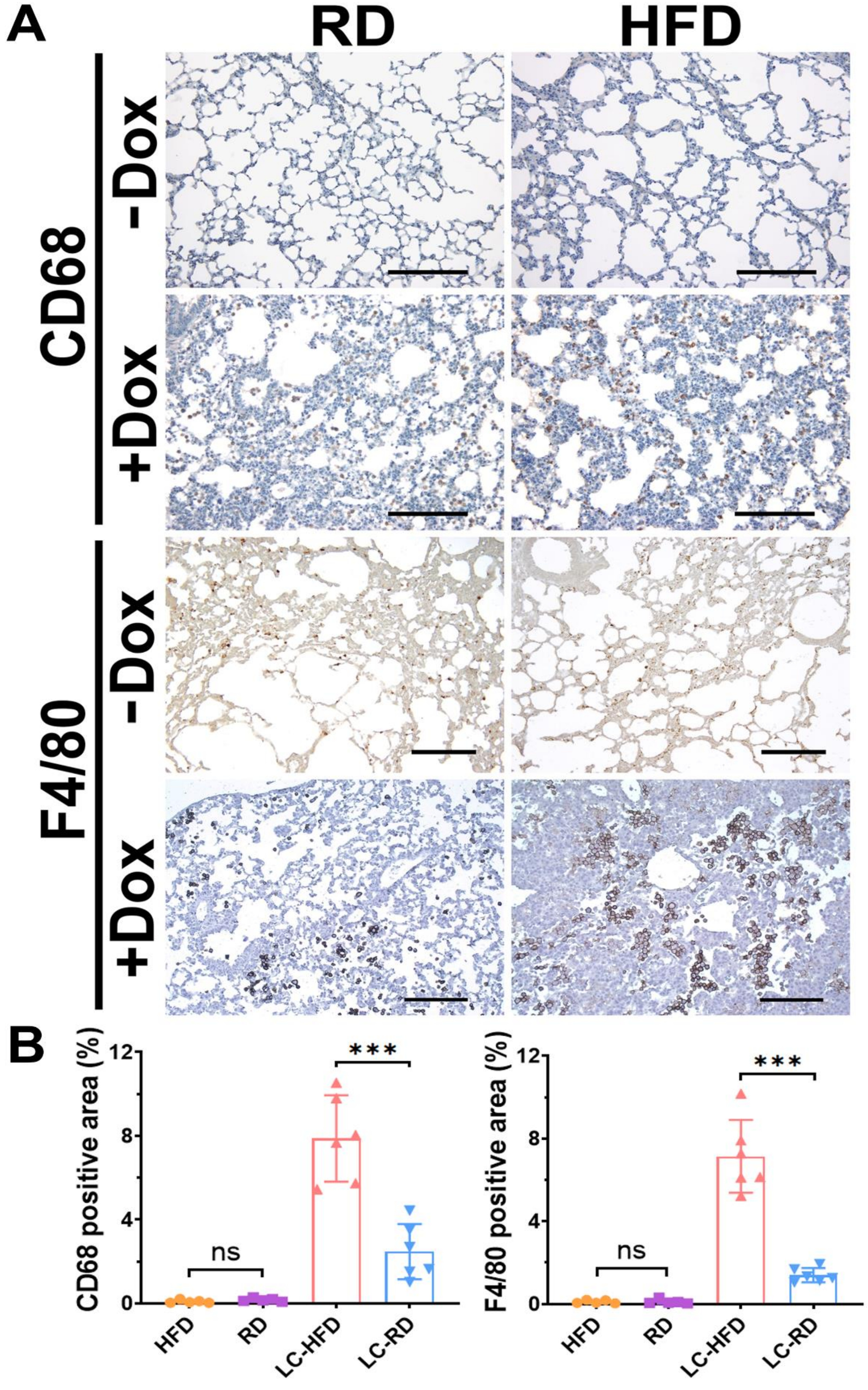

Disease Models & Mechanisms • Supplementary information

Fig. S1. Immunohistochemical staining for macrophages in lung cancer tissues. (A) Lung cancer tissue sections from HFD, RD, LC-HFD, and LC-RD groups were performed immunohistochemical staining by CD68 and F4/80 antibodies. +Dox represents transgenic mice with lung cancer induction by doxycycline treatments (LC) while -Dox represents transgenic mice without doxycycline treatments for lung cancer induction. (B) Quantification of CD68 or F4/80 positive cells. (n=6 for each group). RD, regular diet; HFD, high-fat diet. Scale bar represented 200 μm. Results are shown as mean ± SD, and a two-tailed Student's *t*-test was used for statistical analysis. ***, $p < 0.001$; ns, not significant.

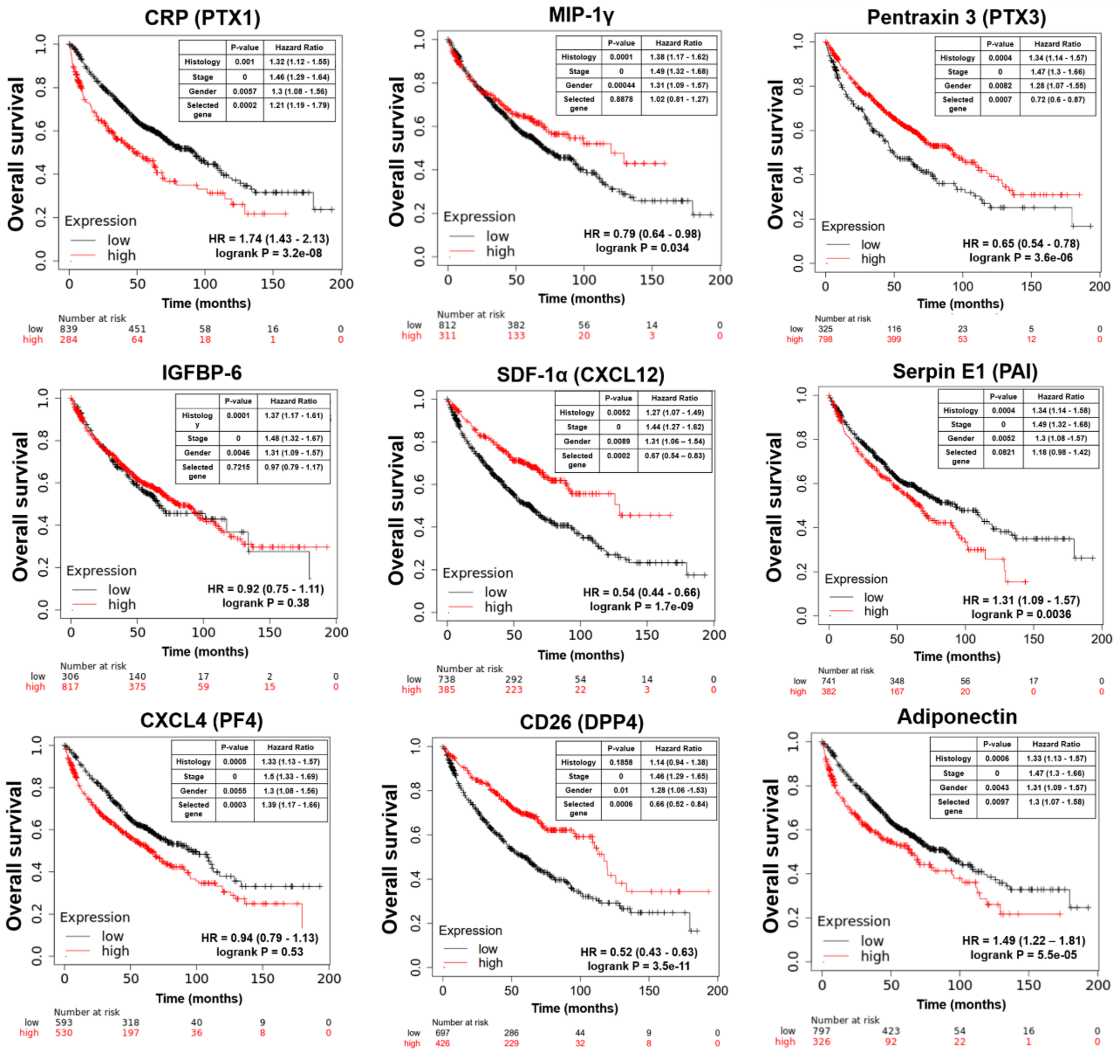

Fig. S2. Kaplan-Meier survival analysis in various cytokines and adipokines expression.

Prognostic prediction power for overall survival of the cytokines and adipokines. The survival data were analyzed using Kaplan-Meier Plotter (http://kmplot.com/analysis/index.php) with multivariate Cox proportional hazards regression analysis. The patients were grouped according to the auto-selection of the best cut-off. The $p<0.05$ was considered statistically significant. HR, hazard ratio.

Disease Models & Mechanisms • Supplementary information

Table S1. A list of differentially expressed genes in high-fat diet (HFD)-induced lung cancer tissue.

Gene Symbol	Probe Set ID	Fold Change (LC-HFD vs. LC-RD)	Regulation (LC-HFD vs. LC-RD)	Corrected p-value	LC-HFD Signal Intensity	LC-RD Signal Intensity
1700056N10Rik	1432356_at	3.007	up	3.02E-05	56.97	18.94
2210409O19Rik	1432755_at	2.636	up	9.77E-04	18.04	6.84
2510017J16Rik	1430393_at	2.375	up	1.81E-03	34.54	14.54
2700038G22Rik	1453745_at	2.533	up	2.19E-03	59.75	23.59
3110045A19Rik	1430940_at	2.132	up	2.53E-04	135.59	63.61
5730405O12Rik	1432843_at	2.002	up	2.53E-04	30.96	15.46
5730458M16Rik	1454375_at	2.492	up	5.66E-04	51.17	20.54
8030497I03Rik	1442824_at	2.211	up	2.15E-04	86.28	39.03
ACE	1451911_a_at	3.737	up	1.49E-04	148.98	39.86
ACTN1	1427385_s_at	4.780	up	1.72E-07	256.50	53.66
ADGRG1	1433485_x_at	2.183	up	3.76E-05	620.04	284.09
ADPGK	1451079_at	2.032	up	1.77E-03	45.43	22.36
AGAP2	1419789_at	2.350	up	1.64E-03	21.23	9.03
AHDC1	1424956_at	2.038	up	7.57E-04	69.98	34.34
AI480526	1435711_at	2.020	up	1.09E-05	75.41	37.33
ANKRD37	1436538_at	2.577	up	8.13E-05	80.87	31.38
ANPEP	1421424_a_at	3.623	up	2.02E-03	108.17	29.86
ANXA11	1418468_at	2.226	up	1.43E-05	513.89	230.87
ANXA6	1415818_at	2.618	up	2.84E-05	581.90	222.28
AP2A1	1460724_at	2.259	up	6.35E-05	153.25	67.83
AP3S2	1422718_at	2.070	up	2.44E-04	82.25	39.73
ARF4	1423053_at	2.093	up	2.25E-06	636.41	304.04
ARG1	1419549_at	6.620	up	4.83E-05	59.45	8.98
ARHGAP10	1426027_a_at	2.036	up	3.55E-03	13.76	6.76
ARHGEF1	1421164_a_at	2.153	up	3.66E-04	461.83	214.54
ARID1A	1417440_at	3.569	up	3.53E-06	368.77	103.33
ASS1	1416239_at	2.127	up	2.23E-03	42.45	19.95
ATF4	1448135_at	2.052	up	8.38E-06	1037.46	505.58

Disease Models & Mechanisms • Supplementary information

ATN1	1421149_a_at	3.514	up	2.89E-04	70.79	20.14
ATP6V0A1	1417632_at	2.674	up	1.88E-06	518.28	193.85
ATP6V0B	1416769_s_at	2.078	up	1.24E-05	877.66	422.28
ATXN2L	1456476_at	2.383	up	1.10E-03	153.22	64.29
AY036118	1421754_at	2.257	up	7.12E-03	354.13	156.89
BAK1	1425716_s_at	2.027	up	1.06E-03	20.72	10.23
BB283564	1442195_at	2.021	up	1.06E-02	57.97	28.69
BCAT2	1425764_a_at	3.157	up	4.14E-08	79.13	25.06
C1QB	1437726_x_at	2.188	up	2.45E-04	261.07	119.30
C430049A07Rik	1454574_at	2.171	up	4.53E-03	12.11	5.58
C78878	1442566_at	2.276	up	7.96E-03	11.06	4.86
CALCOCO1	1428513_at	2.692	up	8.53E-06	202.25	75.12
CASZ1	1459054_at	2.096	up	3.52E-06	119.83	57.17
Ccl9	1417936_at	3.759	up	1.38E-06	336.56	89.53
CCNT1	1419313_at	3.154	up	4.91E-04	189.23	59.99
Cd99	1430514_a_at	2.206	up	1.93E-04	402.25	182.36
CDKL4	1447767_at	2.402	up	1.76E-02	29.98	12.48
CELSR2	1422073_a_at	2.569	up	6.82E-04	24.39	9.49
CEP250	1421512_at	2.375	up	8.89E-05	32.62	13.74
CFL1	1448346_at	5.193	up	4.08E-06	1380.36	265.81
CLCA1	1416306_at	3.114	up	9.14E-03	13.42	4.31
COL1A1	1423669_at	3.594	up	7.30E-07	267.74	74.50
CSF2RB	1450200_s_at	2.108	up	3.19E-05	139.22	66.04
CSTF1	1417117_at	2.044	up	1.48E-02	31.50	15.41
Cux1	1424668_a_at	3.176	up	2.00E-05	73.56	23.16
DDHD1	1445438_at	2.328	up	6.45E-06	138.08	59.31
DLK1	1449939_s_at	2.790	up	2.21E-04	34.42	12.34
DOT1L	1439488_at	2.074	up	1.16E-03	114.65	55.28
DPP8	1428698_at	2.653	up	3.69E-04	99.99	37.69
DUSP23	1453074_at	2.767	up	8.35E-03	34.92	12.62
E2F4	1451480_at	2.118	up	3.90E-04	32.56	15.37
EHD2	1435785_at	2.707	up	4.45E-04	175.01	64.66
EIF4EBP2	1437733_at	3.704	up	3.89E-05	101.20	27.32

Disease Models & Mechanisms • Supplementary information

EIF4G2	1458431_at	2.890	up	1.69E-04	35.58	12.31
Elf5	1419556_at	2.598	up	8.93E-04	75.05	28.89
ELMOD3	1451392_at	2.115	up	7.43E-05	40.37	19.09
EPN1	1427039_at	2.360	up	5.30E-06	193.74	82.10
EPOP	1426980_s_at	2.821	up	6.55E-05	50.30	17.83
Erdr1	1452406_x_at	2.070	up	1.10E-04	684.31	330.51
FBN2	1422831_at	3.838	up	1.16E-04	43.97	11.46
FBP1	1448470_at	2.963	up	3.66E-06	21.51	7.26
FOXP4	1429719_at	2.750	up	1.39E-04	149.65	54.42
GALC	1420547_at	3.163	up	8.82E-05	79.03	24.99
GAS5	1424843_a_at	2.256	up	1.00E-05	192.79	85.46
Gm19325	1438393_at	2.016	up	4.20E-04	21.34	10.59
Gm29673/Gm6958	1426936_at	2.003	up	2.94E-04	127.02	63.41
Gm31462	1459648_at	2.899	up	3.51E-05	34.31	11.84
GPRC5A	1437486_at	3.325	up	1.28E-07	3376.13	1015.34
H3-3A/H3-3B	1430357_at	2.223	up	8.44E-04	91.06	40.96
HM13	1438456_at	2.082	up	1.26E-03	57.96	27.83
HMBOX1	1436341_at	2.409	up	1.83E-03	128.39	53.31
HMG20B	1417637_a_at	2.099	up	1.91E-04	143.94	68.58
HNF1B	1421224_a_at	2.064	up	3.14E-06	46.66	22.61
HNRNPAB	1426114_at	3.210	up	3.42E-05	1247.98	388.79
HOMER2	1424367_a_at	2.004	up	3.99E-04	76.76	38.30
HS6ST1	1417293_at	2.056	up	6.29E-06	181.54	88.31
Hspa1b	1427126_at	2.752	up	8.02E-05	315.92	114.81
HSPA8	1431182_at	2.243	up	2.68E-03	66.14	29.49
HSPG2	1418670_s_at	2.280	up	1.14E-03	203.39	89.22
Igk	1452463_x_at	2.191	up	1.03E-05	402.22	183.58
Igkv1-117	1460423_x_at	2.615	up	1.08E-04	23.36	8.93
Igkv15-103	1427837_at	2.026	up	3.76E-04	12.62	6.23
IKBKB	1445141_at	2.069	up	6.97E-04	31.88	15.41
ITGA3	1421997_s_at	2.967	up	3.02E-07	345.65	116.48
ITGA5	1423267_s_at	3.686	up	5.14E-06	58.48	15.87
JUP	1426873_s_at	2.281	up	1.27E-04	353.05	154.76

Disease Models & Mechanisms • Supplementary information

KCNC3	1440861_a_at	2.564	up	9.66E-06	205.60	80.18
KCNK3	1426058_a_at	2.014	up	3.38E-02	22.62	11.24
KDM2A	1455942_at	2.343	up	6.18E-05	166.40	71.01
KDM5C	1426497_at	3.262	up	1.76E-05	38.97	11.95
KIFC2	1421312_a_at	2.017	up	3.73E-04	47.13	23.37
KLHL18	1455543_at	2.340	up	9.94E-03	55.25	23.61
KNG1	1416676_at	3.846	up	3.74E-05	203.67	52.95
Krtap17-1	1453523_at	2.240	up	5.68E-05	121.11	54.08
LASP1	1456309_x_at	2.135	up	4.36E-05	693.78	324.94
LBH	1429088_at	2.566	up	1.48E-03	151.34	58.98
LENG8	1435152_at	3.777	up	8.98E-06	266.39	70.53
LFNG	1420643_at	2.188	up	1.20E-03	97.13	44.40
LIPG	1421262_at	2.017	up	8.57E-04	61.25	30.37
LRCH4	1424879_at	2.031	up	9.87E-04	111.17	54.75
LRP5	1449299_at	2.550	up	2.82E-04	37.09	14.55
MAN1B1	1452235_at	2.122	up	7.57E-04	193.44	91.17
MAPRE3	1427079_at	2.578	up	1.46E-04	88.32	34.26
MARCHF7	1458690_at	3.002	up	2.33E-05	125.08	41.67
MARK2	1435889_at	3.149	up	2.32E-04	160.39	50.94
MATR3	1458508_at	2.116	up	2.55E-05	47.69	22.54
MAZ	1427099_at	2.995	up	1.77E-04	221.88	74.07
Meg3	1426758_s_at	3.743	up	6.57E-05	374.50	100.04
MICAL2	1455685_at	4.564	up	2.81E-05	125.63	27.53
MID1	1438239_at	4.080	up	7.13E-05	141.79	34.76
Mirg	1457030_at	3.153	up	2.85E-03	30.70	9.74
MLF2	1423916_s_at	2.899	up	5.27E-05	976.11	336.69
MMP12	1449153_at	2.320	up	2.14E-05	179.58	77.42
NAB1	1438819_at	2.004	up	7.17E-03	31.32	15.63
NASP	1444583_at	2.742	up	5.35E-05	27.28	9.95
NAV2	1444706_at	2.103	up	1.50E-04	95.23	45.27
NCSTN	1418570_at	3.403	up	7.31E-05	167.14	49.11
NECTIN1	1438421_at	2.205	up	8.02E-06	40.39	18.32
NEDD4L	1437400_at	3.455	up	8.65E-05	150.85	43.66

Disease Models & Mechanisms • Supplementary information

NEK7	1444753_at	2.840	up	8.20E-04	75.51	26.59
NFIC	1422565_s_at	3.993	up	1.20E-03	30.75	7.70
NFKB2	1429128_x_at	2.129	up	6.17E-04	85.11	39.99
NKX2-1	1422346_at	2.517	up	1.05E-03	94.66	37.60
NNAT	1423506_a_at	4.665	up	2.91E-05	210.03	45.02
NOTCH3	1421964_at	2.015	up	1.25E-02	43.36	21.52
NPNT	1426561_a_at	4.169	up	6.94E-06	254.46	61.04
NPR1	1449160_at	2.399	up	2.82E-04	27.27	11.37
NTRK2	1435196_at	2.087	up	1.82E-04	110.02	52.71
OAS1	1424775_at	2.091	up	2.26E-04	221.10	105.73
OAS2	1425065_at	2.408	up	1.11E-06	77.75	32.29
OAS3	1425374_at	2.220	up	2.74E-02	27.21	12.25
Oasl2	1453196_a_at	2.435	up	4.70E-05	129.88	53.35
OGG1	1430078_a_at	2.080	up	5.44E-04	50.05	24.06
OGT	1436780_at	4.106	up	2.09E-05	152.43	37.13
OLFML3	1428048_at	2.453	up	1.78E-04	22.38	9.12
PABPC1	1453840_at	2.755	up	1.09E-03	87.25	31.67
PATJ	1442244_at	2.108	up	1.79E-04	39.14	18.57
PCGF2	1420645_at	2.619	up	1.52E-04	88.82	33.91
PDK4	1417273_at	2.132	up	1.79E-04	163.84	76.84
PFKL	1439148_a_at	2.949	up	1.25E-05	270.80	91.82
PGLYRP1	1449184_at	2.540	up	7.31E-05	119.96	47.22
PICALM	1446968_at	4.366	up	2.51E-05	101.90	23.34
PIK3CA	1440054_at	2.050	up	1.70E-03	16.70	8.15
PIK3CD	1443798_at	2.087	up	5.29E-04	2758.47	1321.97
PITPNM2	1419757_at	3.151	up	7.02E-05	309.84	98.34
PLA2G7	1430700_a_at	2.170	up	5.50E-05	87.18	40.17
PLEKHJ1	1424179_at	3.695	up	8.28E-04	147.73	39.98
PLTP	1417963_at	3.355	up	1.36E-06	414.50	123.55
POLR2A	1422311_a_at	2.313	up	1.38E-03	50.69	21.91
PPP2R5E	1428462_at	2.573	up	6.65E-05	26.67	10.37
PRKACA	1450519_a_at	2.372	up	9.77E-05	274.44	115.69
PRKCE	1449956_at	2.086	up	1.10E-02	16.04	7.69

Disease Models & Mechanisms • Supplementary information

Prm1	1437054_x_at	2.104	up	1.90E-04	2158.83	1026.28
PROSER2	1425000_s_at	2.164	up	1.92E-04	22.22	10.27
PRRC2A	1422799_at	2.052	up	1.34E-06	427.46	208.28
PSME3	1418078_at	4.008	up	4.63E-05	166.52	41.54
PTPRJ	1427629_at	5.514	up	7.26E-04	64.62	11.72
PYM1	1453315_at	2.002	up	3.48E-03	31.63	15.80
RAVER1	1427110_at	3.421	up	1.01E-04	77.32	22.60
RBM3	1429169_at	3.106	up	4.33E-05	53.14	17.11
RC3H2	1426925_at	2.192	up	2.50E-04	51.93	23.69
RGS5	1417466_at	4.702	up	1.04E-07	408.17	86.81
Rian	1427580_a_at	2.198	up	1.10E-03	56.98	25.93
RICTOR	1441753_at	2.252	up	2.81E-04	137.73	61.16
RIMS2	1422809_at	2.252	up	1.02E-02	40.10	17.81
RNASE2	1425295_at	4.953	up	3.94E-03	25.38	5.12
RPS15A	1457726_at	2.579	up	1.25E-02	26.58	10.31
RSKR	1444656_at	2.196	up	3.93E-03	13.69	6.23
SAFB2	1427987_at	2.852	up	4.04E-04	117.51	41.20
Scaf1	1434908_at	3.283	up	2.74E-04	68.17	20.76
SCAF8	1438419_at	2.020	up	5.69E-05	104.14	51.56
SELENOW	1416521_at	4.951	up	2.41E-04	688.31	139.01
Sf1	1422321_a_at	2.299	up	2.08E-03	78.58	34.18
Sf3a2	1450576_a_at	2.414	up	2.48E-04	72.20	29.91
SFTPC	1438290_x_at	2.814	up	1.15E-02	13.04	4.63
SHANK3	1419137_at	2.054	up	7.23E-05	100.03	48.70
SLBP	1430058_at	2.897	up	1.63E-03	76.01	26.23
SLC25A23	1419045_at	2.199	up	1.17E-05	149.40	67.95
Slc2a4rg-ps	1438296_at	2.241	up	1.19E-02	32.98	14.71
SLC9A3R2	1452976_a_at	2.179	up	9.59E-05	185.20	85.00
SMN1/SMN2	1426596_a_at	2.018	up	3.06E-04	57.17	28.33
SNRNP70	1429009_at	2.151	up	3.52E-04	328.57	152.77
SOD3	1417633_at	2.234	up	1.37E-04	300.37	134.46
SPEN	1420397_a_at	2.135	up	5.10E-04	108.21	50.69
SPOCK2	1435026_at	2.271	up	4.21E-05	522.00	229.90

Disease Models & Mechanisms • Supplementary information

SPP1	1449254_at	2.099	up	4.65E-05	2922.57	1392.23
SPRR1A	1449133_at	2.390	up	1.93E-04	232.05	97.11
SSBP3	1425940_a_at	2.222	up	9.02E-05	118.24	53.21
STRBP	1444001_at	2.391	up	5.10E-04	34.95	14.62
TAF1D	1452987_at	2.822	up	1.83E-05	21.26	7.53
TAOK2	1438208_at	2.089	up	2.18E-04	145.93	69.85
TAOK3	1448019_at	2.560	up	8.56E-04	250.28	97.76
TGFB1	1420653_at	3.918	up	1.71E-06	291.42	74.38
THEM6	1428860_at	2.156	up	1.50E-03	32.37	15.01
TINAGL1	1417109_at	2.090	up	1.33E-04	647.17	309.72
TLE5	1420619_a_at	3.414	up	2.95E-08	1096.74	321.25
TMEM106C	1434783_at	2.482	up	2.36E-04	70.76	28.51
Tmsb4x (includes others)	1417219_s_at	3.052	up	5.65E-07	1157.54	379.32
TOMM6	1423087_a_at	2.751	up	5.12E-06	959.21	348.65
TP53	1427739_a_at	2.290	up	3.41E-04	62.22	27.18
Trim30a/Trim30d	1438716_at	2.209	up	8.97E-06	38.08	17.24
UBQLN2	1420844_at	2.121	up	1.99E-02	24.21	11.42
UCP2	1459741_x_at	2.095	up	2.32E-05	674.52	321.91
USP15	1442307_at	2.131	up	2.63E-03	42.81	20.09
USP3	1441056_at	2.493	up	3.44E-04	52.76	21.17
VAT1	1423726_at	2.266	up	8.47E-05	153.33	67.68
WDR83OS	1424117_at	2.011	up	3.85E-05	281.38	139.93
WIZ	1418202_a_at	2.577	up	1.21E-05	133.00	51.61
ZFHX3	1429725_at	2.031	up	1.55E-04	92.38	45.47
ZFP57	1450929_at	4.056	up	3.28E-05	81.25	20.03
ZFR	1449552_at	2.333	up	1.93E-05	110.84	47.51
ZNF385A	1418865_at	2.082	up	8.05E-04	47.64	22.88
ZNF395	1452336_at	3.129	up	6.15E-05	69.25	22.13
1810014B01Rik	1430991_at	12.823	down	6.89E-06	13.08	167.70
2410022M11Rik	1453081_at	2.131	down	1.73E-03	34.20	72.88
2610021A01Rik	1438786_a_at	2.300	down	6.99E-03	17.32	39.84
2610044O15Rik8	1428693_at	2.217	down	2.02E-02	12.51	27.75

Disease Models & Mechanisms • Supplementary information

2810416G20Rik	1431220_at	2.357	down	6.26E-04	5.26	12.40
4930422N03Rik	1453375_at	2.027	down	1.57E-03	4.98	10.10
4930432E11Rik/4932431P20Rik	1432882_at	2.036	down	3.72E-04	50.67	103.17
9530006C21Rik	1441779_at	2.590	down	1.66E-04	49.93	129.32
A130038J17Rik	1446619_at	10.469	down	1.06E-06	9.56	100.13
A630026N12Rik	1436447_at	2.776	down	1.16E-05	15.37	42.67
AA617406	1443592_at	2.134	down	1.28E-03	18.16	38.75
Abi3bp	1427054_s_at	2.696	down	4.70E-04	29.01	78.20
ACKR4	1437668_at	2.643	down	3.00E-04	14.43	38.15
ACTL6A	1429883_at	2.025	down	2.99E-04	41.04	83.08
ADRB2	1437302_at	2.010	down	2.78E-04	111.23	223.63
AGMO	1434191_at	2.628	down	6.91E-05	62.87	165.23
Airn	1436627_at	2.019	down	4.16E-03	26.82	54.15
ALDH1L1	1424400_a_at	2.093	down	1.40E-04	49.49	103.59
ALDH2	1434987_at	11.252	down	1.03E-03	5.29	59.55
AMN1	1441141_at	2.384	down	5.56E-04	24.00	57.23
ANKRD1	1420992_at	2.074	down	4.11E-03	12.83	26.61
ANXA1	1444016_at	3.077	down	9.82E-03	7.67	23.62
Apoc1	1417561_at	2.093	down	3.45E-04	137.49	287.79
ARL4A	1435092_at	3.879	down	3.90E-04	8.04	31.17
ARMC8	1434949_at	2.096	down	4.59E-04	24.31	50.96
ARPC2	1442295_at	3.185	down	2.95E-04	6.98	22.22
ARRDC3	1454617_at	3.220	down	7.73E-06	102.59	330.30
ASPM	1422814_at	2.082	down	1.45E-04	35.18	73.25
ATP7B	1436481_at	2.732	down	4.95E-04	50.81	138.82
ATR	1438921_at	2.046	down	9.32E-03	6.67	13.65
ATRX	1438750_at	2.047	down	5.35E-05	31.18	63.84
ATXN10	1431377_at	2.818	down	2.98E-03	11.75	33.12
AU015263	1439819_at	5.024	down	9.47E-07	31.79	159.73
BANK1	1456328_at	2.024	down	8.35E-03	12.04	24.36
BEX1	1417388_at	2.061	down	3.35E-05	102.86	211.99
BRCA1	1424629_at	2.131	down	1.38E-03	24.14	51.43

Disease Models & Mechanisms • Supplementary information

C2CD4B	1453326_at	2.378	down	1.24E-04	55.47	131.93
CACNA2D1	1440397_at	2.089	down	1.18E-02	7.12	14.87
CCDC122	1458438_at	2.418	down	4.00E-05	61.98	149.86
CCDC34	1449345_at	2.275	down	3.39E-03	16.18	36.81
CDCA8	1436847_s_at	2.125	down	1.00E-03	45.84	97.40
CENPI	1436723_at	2.008	down	2.73E-03	11.38	22.84
CENPK	1418264_at	2.384	down	1.43E-05	20.68	49.32
CENPP	1432361_a_at	2.282	down	5.66E-04	12.67	28.92
CENPQ	1423620_at	2.329	down	2.09E-04	30.92	72.02
CEP290	1425642_at	2.088	down	1.23E-02	14.32	29.90
CEP55	1452242_at	2.097	down	8.91E-05	79.74	167.20
Chaserr	1452991_at	2.686	down	2.22E-03	17.51	47.02
CHKA	1442277_at	2.185	down	8.89E-04	59.06	129.05
CIRBP	1416332_at	2.527	down	1.58E-05	110.86	280.14
Cldn34c1 (includes others)	1439279_at	2.601	down	9.94E-05	11.49	29.88
CORO1C	1419911_at	2.105	down	2.39E-04	9.76	20.55
Cox7c	1459885_s_at	2.447	down	2.46E-03	13.07	31.97
CSF2	1427429_at	3.065	down	8.69E-04	19.40	59.45
CTBS	1429943_at	2.120	down	1.81E-03	34.80	73.76
Ctla2a/Ctla2b	1452352_at	2.105	down	1.45E-03	35.90	75.57
CWF19L2	1456019_at	2.169	down	8.86E-05	90.01	195.18
CXCL2	1419209_at	2.930	down	1.62E-03	10.63	31.14
CYP2J2	1440691_at	2.032	down	3.55E-03	13.99	28.43
CYP51A1	1422533_at	2.385	down	4.09E-04	28.98	69.11
CYRIB	1436088_at	2.103	down	3.93E-04	21.07	44.31
CYTH3	1446268_at	2.959	down	2.16E-03	5.38	15.92
D10Bwg1070e	1439053_at	2.178	down	1.01E-02	11.80	25.69
D530037H12Rik	1437917_at	2.777	down	3.36E-05	19.61	54.45
D730035F11Rik	1457163_at	2.238	down	1.48E-04	12.06	26.99
D9Wsu90e	1442409_at	2.344	down	7.27E-03	19.19	44.99
DDIT4	1428306_at	2.247	down	5.96E-08	141.48	317.95
Dep1	1438892_at	3.372	down	1.60E-03	14.85	50.07

Disease Models & Mechanisms • Supplementary information

DEPDC1	1424292_at	2.763	down	1.45E-03	11.04	30.50
DEPP1	1433836_a_at	2.012	down	6.76E-03	102.23	205.66
DHX30	1453251_at	2.478	down	3.36E-03	8.79	21.77
DSCC1	1452912_at	2.838	down	5.37E-04	6.51	18.49
DTL	1434695_at	2.106	down	1.04E-03	16.55	34.86
DUSP1	1448830_at	2.895	down	1.21E-05	411.20	1190.34
DYNC2H1	1429202_at	2.121	down	6.14E-04	18.53	39.30
E030016H06Rik	1440443_at	4.133	down	6.57E-06	14.95	61.79
ECT2	1419513_a_at	2.271	down	1.10E-03	55.68	126.46
EFCAB7	1451349_at	2.158	down	1.45E-03	8.39	18.11
EGR2	1427683_at	2.296	down	6.68E-05	22.66	52.03
EML5	1437791_s_at	2.536	down	1.28E-03	16.33	41.41
ERLIN1	1441344_at	2.070	down	8.11E-04	8.88	18.37
ESCO2	1428304_at	2.602	down	3.00E-04	14.29	37.18
ETFBKMT	1457915_at	2.434	down	4.81E-05	24.92	60.66
FAM162B	1453496_at	2.273	down	3.17E-04	20.59	46.80
FEM1C	1438075_at	3.029	down	4.36E-04	18.28	55.38
FIGNL1	1422430_at	2.524	down	5.39E-03	19.35	48.86
Fip1l1	1443586_at	2.039	down	3.55E-03	24.17	49.28
FNIP1	1419924_at	3.693	down	1.06E-04	9.55	35.26
FOS	1423100_at	4.081	down	1.29E-07	149.50	610.11
FYCO1	1453424_at	2.672	down	2.35E-03	18.28	48.83
G0S2	1448700_at	2.270	down	1.67E-06	35.25	80.01
G2E3	1455355_at	2.114	down	1.07E-03	13.29	28.10
GADD45G	1453851_a_at	2.989	down	8.45E-05	67.26	201.04
GDPGP1	1444106_at	2.072	down	1.76E-03	11.47	23.76
Gimap9	1437756_at	2.247	down	5.03E-03	10.43	23.44
Gm14419 (includes others)	1434097_at	3.384	down	7.87E-05	17.33	58.64
Gm19439	1435119_at	2.050	down	4.45E-05	61.07	125.20
Gm46901	1444673_at	2.215	down	3.90E-02	7.06	15.64
Gm5817	1435198_at	2.200	down	3.24E-04	36.65	80.64
GOLPH3	1420117_at	2.396	down	9.21E-04	23.38	56.02

Disease Models & Mechanisms • Supplementary information

GPCPD1	1429639_at	4.450	down	5.58E-06	33.13	147.41
GPM6A	1426442_at	2.104	down	6.86E-04	46.24	97.32
GPR34	1422542_at	2.059	down	4.43E-04	6.05	12.46
GSTA3	1423436_at	2.591	down	2.00E-05	72.28	187.26
GSTA5	1421040_a_at	2.133	down	6.90E-03	6.96	14.86
GTF2H2	1450701_a_at	2.008	down	1.01E-04	40.39	81.12
GULP1	1434423_at	2.571	down	1.86E-03	24.24	62.33
H2AC6	1438009_at	2.321	down	1.01E-07	598.96	1390.37
HASPIN	1450886_at	2.041	down	6.45E-04	6.39	13.04
HELLS	1417541_at	2.148	down	6.74E-03	21.74	46.69
HERC1	1425378_at	2.410	down	8.11E-05	5.78	13.93
HLA-A	1418734_at	2.317	down	3.63E-03	37.57	87.06
HMGB2	1438307_at	2.317	down	1.11E-04	10.03	23.23
HMGCS1	1441536_at	2.123	down	3.76E-03	4.62	9.81
HOATZ	1430762_at	2.105	down	2.94E-03	13.54	28.49
HPGD	1419906_at	3.036	down	1.15E-03	22.80	69.22
ID1	1425895_a_at	2.096	down	7.39E-06	174.84	366.47
ID2	1453596_at	6.638	down	4.42E-05	6.82	45.27
IDI1	1423804_a_at	2.016	down	1.12E-02	49.59	99.96
INSIG1	1454671_at	2.161	down	1.67E-06	116.44	251.67
ITGA8	1427489_at	2.092	down	4.46E-04	25.45	53.24
IVNS1ABP	1420961_a_at	2.039	down	3.55E-04	21.98	44.82
KATNBL1	1443314_at	2.163	down	1.39E-04	17.27	37.35
KBTBD7	1428192_at	2.217	down	3.82E-03	16.21	35.94
KCNJ15	1443054_at	2.597	down	1.84E-05	35.27	91.58
KDM2B	1459861_s_at	2.134	down	9.30E-04	83.01	177.17
KIAA0040	1456446_at	2.421	down	4.62E-06	46.38	112.29
KIF16B	1429063_s_at	2.027	down	4.56E-04	75.18	152.38
KLF5	1451739_at	2.082	down	1.00E-03	31.55	65.70
LCMT2	1433518_at	2.384	down	3.49E-04	32.02	76.35
LCORL	1455260_at	2.916	down	1.78E-04	14.72	42.94
LIN54	1435181_at	2.036	down	1.36E-03	34.03	69.28
LRAT	1444487_at	4.476	down	1.30E-04	13.92	62.33

Disease Models & Mechanisms • Supplementary information

LY6E	1439773_at	2.739	down	3.51E-05	76.66	209.94
LYVE1	1429379_at	3.207	down	3.71E-07	115.41	370.10
MALAT1	1436202_at	3.738	down	3.69E-05	61.04	228.15
MAPK6	1429963_at	2.537	down	5.33E-03	14.29	36.25
Mapre2	1451989_a_at	2.258	down	7.00E-05	423.40	956.23
MBD1	1453678_at	2.522	down	3.40E-03	5.61	14.16
MECP2	1438930_s_at	2.342	down	1.03E-05	29.96	70.15
MELK	1416558_at	2.108	down	2.87E-03	33.99	71.66
MORF4L1	1437801_at	3.169	down	3.09E-04	25.84	81.88
MSL2	1429108_at	2.119	down	1.22E-04	36.46	77.26
Mt1	1451612_at	3.281	down	1.83E-03	11.77	38.62
MYL7	1449071_at	2.345	down	7.43E-06	14.70	34.45
MYO1H	1450543_at	3.716	down	7.01E-05	12.42	46.14
N4BP2L1	1417707_at	2.029	down	1.14E-04	39.35	79.85
NAP1L3	1418500_at	2.086	down	4.62E-03	5.76	12.01
NCAPG2	1417926_at	2.249	down	3.47E-04	64.07	144.09
NDN	1435382_at	2.229	down	9.45E-04	17.02	37.92
NEK2	1437580_s_at	2.163	down	6.35E-04	46.65	100.91
NFE2	1452001_at	2.259	down	3.05E-03	11.13	25.16
NFE2L2	1457117_at	3.047	down	8.82E-05	18.06	55.02
NFKBIZ	1457404_at	2.195	down	1.90E-04	23.02	50.53
NMRK1	1425646_at	2.075	down	3.68E-03	6.47	13.42
NOL11	1439166_at	2.027	down	3.30E-03	5.97	12.10
NPR3	1435184_at	2.351	down	9.85E-04	27.19	63.92
NQO1	1423627_at	2.284	down	1.33E-02	12.88	29.42
NRP1	1457198_at	2.300	down	1.10E-04	71.86	165.29
NSUN3	1436735_at	2.242	down	4.46E-04	38.67	86.69
NTF3	1434802_s_at	2.050	down	3.58E-03	19.43	39.84
NUDT12	1453139_at	2.049	down	2.96E-03	46.26	94.76
NUS1	1419915_at	2.620	down	9.44E-04	13.13	34.39
NUSAP1	1416309_at	2.216	down	1.41E-04	66.64	147.70
OGN	1419662_at	2.836	down	6.95E-06	64.89	184.02
Particl	1456779_a_at	2.117	down	5.13E-02	9.44	19.99

PBK	1448627_s_at	2.185	down	3.90E-04	111.64	243.91
PDP1	1438201_at	2.045	down	2.60E-03	30.65	62.70
PER3	1441445_at	2.072	down	8.40E-05	42.54	88.15
PEX13	1422472_at	2.407	down	2.15E-04	6.93	16.68
PHEX	1421979_at	2.164	down	5.06E-03	9.67	20.94
PHF20L1	1454939_at	2.124	down	8.02E-05	17.79	37.79
PID1	1436999_at	2.072	down	1.39E-04	62.49	129.50
POLR3A	1437525_a_at	2.011	down	2.20E-03	43.80	88.08
PPARGC1A	1434099_at	2.108	down	8.50E-03	14.79	31.17
Ppbp	1418480_at	4.567	down	9.40E-07	24.52	112.00
PPP1CB	1431328_at	2.270	down	5.53E-05	22.50	51.07
PPP2R2D	1420034_at	2.101	down	3.10E-04	8.13	17.08
PRDM2	1429251_at	2.504	down	2.26E-04	28.55	71.48
PRDX6	1442878_at	2.617	down	5.49E-04	19.50	51.03
PRMT8	1435204_at	2.670	down	8.04E-05	93.93	250.83
PRSS23	1437671_x_at	2.252	down	6.47E-05	40.37	90.90
PRXL2A	1447774_x_at	2.073	down	5.94E-04	36.87	76.46
PSMA6	1435316_at	2.364	down	2.02E-03	4.04	9.55
PTPRC	1440165_at	2.388	down	5.32E-02	6.30	15.05
Ptprd	1435537_at	2.175	down	7.66E-04	46.47	101.07
PTPRN2	1435968_at	2.218	down	6.71E-04	54.23	120.25
PTTG1IP	1420132_s_at	2.016	down	1.06E-03	8.96	18.06
PXYLP1	1456735_x_at	2.379	down	1.18E-03	65.72	156.36
R3HDM1	1439572_at	2.248	down	2.36E-03	13.60	30.56
RAB2A	1419946_s_at	4.687	down	3.51E-04	9.28	43.48
RAB40B	1436566_at	2.006	down	3.23E-03	8.31	16.67
RANBP6	1435167_at	2.295	down	3.96E-03	24.89	57.13
RASD1	1423619_at	2.214	down	4.27E-03	13.22	29.26
RBBP6	1425115_at	2.296	down	8.58E-05	21.19	48.66
RBL1	1425166_at	2.034	down	2.96E-03	19.27	39.20
RBM39	1420982_at	2.429	down	8.02E-05	51.86	125.97
RGPD4 (includes others)	1440104_at	3.188	down	6.85E-04	8.99	28.65

Disease Models & Mechanisms • Supplementary information

RGS1	1417601_at	5.127	down	8.92E-06	14.75	75.63
RGS18	1420398_at	3.730	down	4.90E-04	4.91	18.31
RGS2	1419248_at	2.215	down	5.65E-06	101.36	224.50
RMDN1	1418658_at	2.116	down	3.13E-04	59.16	125.17
Rn18s	AFFX-18SRNAMur/X00686_M_at	38.201	down	2.34E-06	141.57	5408.20
RNF186	1424794_at	2.359	down	4.22E-06	77.93	183.83
RPL27A	1437729_at	2.992	down	4.14E-04	68.28	204.31
RPRD2	1434817_s_at	3.679	down	2.06E-04	21.62	79.55
RPS6KA5	1431050_at	2.952	down	1.45E-03	14.29	42.18
RRM2	1448226_at	2.108	down	3.51E-04	61.72	130.09
SANBR	1428652_at	2.047	down	2.61E-03	94.45	193.37
SCD	1415965_at	2.548	down	1.16E-04	365.96	932.35
SCEL	1422837_at	2.088	down	8.49E-05	74.55	155.66
SCN3A	1439204_at	2.983	down	9.53E-03	7.33	21.85
SETD5	1439515_at	2.355	down	1.56E-04	19.15	45.10
SFT2D3	1435278_at	2.195	down	1.81E-04	23.39	51.36
SGK1	1416041_at	2.542	down	8.56E-05	461.85	1174.19
SLC16A7	1448502_at	2.031	down	8.73E-04	35.63	72.37
SLC25A53	1453040_at	2.001	down	4.55E-03	9.33	18.66
SLC2A3	1437052_s_at	2.116	down	2.26E-03	19.07	40.35
SLC38A4	1428111_at	3.342	down	2.73E-04	26.03	87.00
SLC38A5	1454622_at	2.064	down	3.40E-04	21.59	44.55
SLC4A1	1434502_x_at	2.456	down	2.53E-03	8.47	20.80
SNCA	1436853_a_at	2.483	down	3.71E-04	32.66	81.10
Snhg11	1434292_at	2.293	down	4.45E-03	28.06	64.35
SNRNP40	1452713_a_at	2.350	down	1.15E-04	136.10	319.84
SOCS2	1446085_at	11.562	down	2.72E-07	8.49	98.21
SOX2	1416967_at	2.131	down	2.47E-04	22.43	47.82
SPAG5	1433893_s_at	2.152	down	1.22E-02	11.64	25.05
SPTLC2	1454257_at	2.007	down	6.83E-03	22.30	44.76
SQLE	1415993_at	2.046	down	1.17E-05	117.30	240.06
SRSF11	1430077_at	2.521	down	8.07E-05	21.46	54.10

Disease Models & Mechanisms • Supplementary information

SRSF3	1416151_at	2.189	down	2.99E-04	106.19	232.44
SSPN	1417644_at	2.280	down	3.70E-05	53.58	122.16
STK17B	1430165_at	4.079	down	1.96E-04	12.51	51.02
STRAP	1419913_at	2.154	down	1.57E-04	13.93	30.02
STXBP6	1435396_at	2.010	down	1.96E-04	27.30	54.86
TAF7	1423169_at	2.075	down	1.37E-03	14.24	29.56
TASP1	1451998_at	2.177	down	3.06E-03	8.32	18.10
TAX1BP1	1420175_at	2.523	down	3.42E-03	18.66	47.08
TC2N	1451838_a_at	2.144	down	3.89E-03	61.92	132.78
TCEANC	1435236_at	2.201	down	5.31E-05	33.02	72.69
TDRD3	1434869_at	2.535	down	2.10E-04	33.95	86.08
TFPI	1438530_at	2.005	down	9.41E-05	30.41	60.99
THOC1	1420043_s_at	2.223	down	4.59E-03	5.56	12.36
TM7SF2	1460684_at	2.154	down	7.70E-05	36.55	78.73
TMED7	1419918_at	2.135	down	1.45E-03	82.61	176.38
TMEM14A	1428447_at	2.053	down	6.45E-04	29.41	60.38
TMX3	1419926_at	3.215	down	2.15E-05	6.30	20.25
TOX3	1436600_at	2.210	down	5.65E-05	24.86	54.94
TRAPPC13	1453332_at	2.450	down	2.93E-04	10.05	24.62
TRIM23	1456089_at	2.302	down	7.87E-04	12.07	27.79
TRIP13	1429295_s_at	2.074	down	8.03E-04	21.33	44.24
TTC30A	1424012_at	2.409	down	1.14E-02	8.38	20.19
TTC30B	1423672_at	2.464	down	3.43E-04	41.58	102.47
TUBB1	1444214_at	2.408	down	9.69E-04	23.68	57.03
USP40	1458079_at	2.704	down	2.77E-06	13.00	35.15
VAMP7	1452007_at	2.026	down	1.12E-03	92.93	188.27
VSTM2A	1452065_at	2.042	down	5.28E-03	16.22	33.12
WDR5B	1453058_at	2.084	down	5.21E-04	13.61	28.36
Xlr4c (includes others)	1449347_a_at	3.249	down	2.71E-05	42.08	136.75
YY2	1437495_at	2.180	down	7.80E-03	16.12	35.14
ZBTB16	1442025_a_at	2.197	down	1.03E-04	67.96	149.32
ZBTB25	1453175_at	2.131	down	1.46E-03	10.15	21.62
ZC3H6	1428682_at	3.489	down	2.82E-03	10.26	35.80

Disease Models & Mechanisms • Supplementary information

ZDHHC20	1431218_at	2.112	down	4.52E-03	12.84	27.12
Zfp119b	1442739_at	2.367	down	1.68E-05	9.08	21.48
Zfp229	1438349_at	2.787	down	1.24E-03	22.67	63.18
Zfp40	1421251_at	2.618	down	6.58E-03	9.85	25.78
Zfp429 (includes others)	1455292_x_at	2.184	down	1.61E-04	30.78	67.21
Zfp472	1425058_at	2.712	down	8.58E-05	27.73	75.23
Zfp58	1455945_at	2.038	down	3.57E-03	17.05	34.75
ZFP62	1425495_at	2.118	down	1.45E-02	24.47	51.84
Zfp719	1436594_at	2.075	down	6.93E-04	24.93	51.74
Zfp748	1456324_at	3.340	down	6.13E-05	15.43	51.56
Zfp763	1434675_at	2.019	down	5.36E-05	38.44	77.62
Zfp781	1445274_at	2.237	down	1.39E-03	6.38	14.28
Zfp788	1429371_at	2.202	down	6.85E-04	21.95	48.33
Zfp81	1444076_at	2.177	down	5.18E-03	15.74	34.27
Zfp955a/Zfp955b	1453198_at	2.236	down	4.91E-04	20.76	46.40
Zfp958	1460369_at	2.893	down	3.75E-04	20.00	57.86
Zfp960/Zfp97	1449972_s_at	2.362	down	8.13E-06	61.44	145.13
Zfp989/Znf41-ps	1436574_at	2.087	down	2.38E-03	24.53	51.19
Zfp994	1444589_at	2.311	down	9.45E-05	36.55	84.49
ZMYM2	1428941_at	2.673	down	1.21E-04	18.14	48.49
ZNF253	1424752_x_at	2.297	down	1.33E-04	54.43	125.01
ZNF35	1460252_s_at	2.541	down	4.19E-03	13.54	34.40
ZNF383	1453212_at	2.393	down	3.18E-05	22.91	54.82
ZNF416	1424974_at	2.029	down	1.09E-04	11.36	23.06
ZNF442	1429712_at	2.208	down	3.64E-03	29.05	64.13
ZNF443	1451518_at	2.233	down	1.44E-03	10.90	24.35
ZNF519	1421139_a_at	2.351	down	2.60E-04	42.97	101.03
ZNF597	1460589_at	2.029	down	4.51E-03	15.78	32.03
ZNF616	1424784_at	2.253	down	4.41E-04	28.48	64.16
ZNF717	1441198_at	2.080	down	1.56E-03	18.16	37.79
ZNF729	1440125_at	2.129	down	7.26E-03	5.09	10.84
ZNF761	1424706_at	2.398	down	6.38E-04	22.87	54.86
ZNF799	1437873_at	3.677	down	3.41E-04	15.96	58.67
ZNF800	1456574_at	2.655	down	2.09E-03	16.46	43.72
ZNF91	1429473_at	2.143	down	4.15E-04	67.15	143.92

Table S2. Results of IPA analysis for potential signaling pathways investigation.[a]

Ingenuity Canonical Pathways	-log (p-value)	z-score	Down-regulated	No change	Up-regulated	No overlap with dataset	Molecules
PI3K/AKT Signaling	3.24	2.646	78/200 (39%)	0/200 (0%)	115/200 (57%)	7/200 (4%)	CSF2RB, IKBKB, ITGA3, ITGA5, ITGA8, NFKB2, PIK3CA ,PIK3CD, PPP2R2D, PPP2R5E, RASD1, TP53
HOTAIR Regulatory Pathway	1.83	2.646	63/163 (39%)	0/163 (0%)	79/163 (48%)	21/163 (13%)	COL1A1, H3-3A/H3-3B, MMP12, NFKB2, PIK3CA, PIK3CD, SPP1, TGFB1
Gαq Signaling	1.31	2.646	71/170 (42%)	0/170 (0%)	88/170 (52%)	11/170 (6%)	IKBKB, NFKB2, PIK3CA, PIK3CD ,PRKCE ,RGS18, RGS2
NF-κB Activation by Viruses	3.09	2.449	33/78 (42%)	0/78 (0%)	44/78 (56%)	1/78 (1%)	IKBKB, ITGA3, NFKB2 ,PIK3CA, PIK3CD, PRKCE, RASD1
PEDF Signaling	2.9	2.449	39/84 (46%)	0/84 (0%)	43/84 (51%)	2/84 (2%)	HNF1B, IKBKB, NFKB2, PIK3CA, PIK3CD, RASD1, TP53
Thrombin Signaling	1.49	2.449	94/225 (42%)	0/225 (0%)	121/225 (54%)	10/225 (4%)	ARHGEF1, IKBKB, MYL7, NFKB2, PIK3CA, PIK3CD, PPP1CB, PRKCE, RASD1
Osteoarthritis Pathway	1.75	2.333	103/236 (44%)	0/236 (0%)	115/236 (49%)	18/236 (8%)	ATF4, DDIT4, ITGA3, ITGA5, ITGA8, MMP12, NFKB2, PPARGC1A, SPP1, TGFB1
Cardiac Hypertrophy Signaling (Enhanced)	1.69	2.324	216/542 (40%)	0/542 (0%)	296/542 (55%)	30/542 (6%)	ACE, ADRB2, CACNA2D1, CSF2, CSF2RB, IKBKB, ITGA3, ITGA5, ITGA8, NFKB2, NPR1, PIK3CA, PIK3CD, PRKACA, PRKCE, RASD1, RPS6KA5, TGFB1
ERK/MAPK Signaling	4.65	2.309	87/215 (40%)	0/215 (0%)	117/215 (54%)	11/215 (5%)	ATF4, DUSP1, FOS, H3-3A/H3-3B, ITGA3, ITGA5, ITGA8, PIK3CA, PIK3CD, PPP1CB, PPP2R5E, PRKACA, PRKCE, RASD1, RPS6KA5

Disease Models & Mechanisms • Supplementary information

Role of Pattern Recognition Receptors in Recognition of Bacteria and Viruses	3	2.236	53/156 (34%)	0/156 (0%)	83/156 (53%)	20/156 (13%)	C1QB, CSF2, NFKB2, OAS1, OAS2, OAS3, PIK3CA, PIK3CD, PRKCE, TGFB1
Paxillin Signaling	2.3	2.236	46/107 (43%)	0/107 (0%)	56/107 (52%)	5/107 (5%)	ACTN1, ITGA3, ITGA5, ITGA8, PIK3CA, PIK3CD, RASD1
Nitric Oxide Signaling in the Cardiovascular System	2.04	2.236	45/120 (38%)	0/120 (0%)	64/120 (53%)	11/120 (9%)	CACNA2D1, KNG1, NPR1, PIK3CA, PIK3CD, PRKACA, PRKCE
Type II Diabetes Mellitus Signaling	1.52	2.236	52/153 (34%)	0/153 (0%)	93/153 (61%)	8/153 (5%)	CACNA2D1, IKBKB, NFKB2, PIK3CA, PIK3CD, PRKCE, SOCS2
Role Of Osteoclasts In Rheumatoid Arthritis Signaling Pathway	1.39	2.111	108/309 (35%)	0/309 (0%)	178/309 (58%)	23/309 (7%)	ATF4, COL1A1, CSF2, FOS,IKBKB, ITGA5, MMP12, PIK3CA, PIK3CD, RASD1, SPP1
Regulation of eIF4 and p70S6K Signaling	4.24	2	72/181 (40%)	0/181 (0%)	90/181 (50%)	19/181 (10%)	EIF4EBP2, EIF4G2, ITGA3, ITGA5, ITGA8, PABPC1, PIK3CA, PIK3CD, PPP2R2D, PPP2R5E, RASD1, Rn18s, RPS15A
CDK5 Signaling	2.73	2	45/115 (39%)	0/115 (0%)	65/115 (57%)	5/115 (4%)	ITGA3, MAPK6, NTRK2, PPP1CB, PPP2R2D, PPP2R5E, PRKACA, RASD1
Small Cell Lung Cancer Signaling	2.57	2	54/96 (56%)	0/96 (0%)	40/96 (42%)	2/96 (2%)	E2F4, IKBKB, NFKB2, PIK3CA, PIK3CD, RBL1, TP53
Pancreatic Adenocarcinoma Signaling	1.93	2	57/126 (45%)	0/126 (0%)	64/126 (51%)	5/126 (4%)	E2F4, NFKB2, PIK3CA, PIK3CD, RBL1, TGFB1, TP53
Macropinocytosis Signaling	1.79	2	33/76 (43%)	0/76 (0%)	41/76 (54%)	2/76 (3%)	ITGA5, PIK3CA, PIK3CD, PRKCE, RASD1
Dopamine-DARPP32 Feedback in cAMP Signaling	1.53	2	67/186 (36%)	0/186 (0%)	109/186 (59%)	10/186 (5%)	ATF4, CACNA2D1, KCNJ15, PPP1CB, PPP2R2D, PPP2R5E, PRKACA, PRKCE
Induction of	1.42	2	27/65 (42%)	0/65	37/65 (57%)	1/65 (2%)	BAK1, IKBKB, NFKB2, TP53

Disease Models & Mechanisms • Supplementary information

Apoptosis by HIV1				(0%)			
IL-17A Signaling in Airway Cells	1.38	2	27/67 (40%)	0/67 (0%)	34/67 (51%)	6/67 (9%)	IKBKB, NFKB2, PIK3CA, PIK3CD
Apelin Adipocyte Signaling Pathway	1.49	-2	41/91 (45%)	0/91 (0%)	42/91 (46%)	8/91 (9%)	GSTA5, MAPK6, PPARGC1A, PRDX6, PRKACA
Superpathway of Cholesterol Biosynthesis	3.66	-2.236	22/29 (76%)	0/29 (0%)	6/29 (21%)	1/29 (3%)	CYP51A1, HMGCS1, IDI1, SQLE, TM7SF2
Semaphorin Neuronal Repulsive Signaling Pathway	3.75	-2.53	59/150 (39%)	0/150 (0%)	79/150 (53%)	12/150 (8%)	CFL1,ITGA3, ITGA5, ITGA8, MYL7, NRP1, PIK3CA, PIK3CD, PPP1CB, PRKACA, TP53
PTEN Signaling	2.54	-2.646	60/151 (40%)	0/151 (0%)	86/151 (57%)	5/151 (3%)	IKBKB, ITGA3, ITGA5, ITGA8, NFKB2, NTRK2, PIK3CA, PIK3CD, RASD1

[a]The genes from cDNA expression array a with fold changes of more or less than two were analyzed by IPA software for potential signaling pathways prediction. Twenty-six significant canonical pathways were identified based on the criteria p-value < 0.05 and z-score > 2 or < -2.

Disease Models & Mechanisms • Supplementary information
